# A Bioinformatic Strategy for the Detection, Classification and Analysis of Bacterial Autotransporters

**DOI:** 10.1371/journal.pone.0043245

**Published:** 2012-08-14

**Authors:** Nermin Celik, Chaille T. Webb, Denisse L. Leyton, Kathryn E. Holt, Eva Heinz, Rebecca Gorrell, Terry Kwok, Thomas Naderer, Richard A. Strugnell, Terence P. Speed, Rohan D. Teasdale, Vladimir A. Likić, Trevor Lithgow

**Affiliations:** 1 Department of Biochemistry and Molecular Biology, Monash University, Clayton, Australia; 2 Department of Microbiology, Monash University, Clayton, Australia; 3 Department of Microbiology and Immunology, The University of Melbourne, Parkville, Australia; 4 Bioinformatics Division, Walter and Eliza Hall Institute, Parkville, Australia; 5 Institute for Molecular Bioscience, The University of Queensland, St. Lucia, Australia; 6 The Bio21 Molecular Science and Biotechnology Institute, The University of Melbourne, Parkville, Australia; University of Georgia, United States of America

## Abstract

Autotransporters are secreted proteins that are assembled into the outer membrane of bacterial cells. The passenger domains of autotransporters are crucial for bacterial pathogenesis, with some remaining attached to the bacterial surface while others are released by proteolysis. An enigma remains as to whether autotransporters should be considered a class of secretion system, or simply a class of substrate with peculiar requirements for their secretion. We sought to establish a sensitive search protocol that could identify and characterize diverse autotransporters from bacterial genome sequence data. The new sequence analysis pipeline identified more than 1500 autotransporter sequences from diverse bacteria, including numerous species of Chlamydiales and Fusobacteria as well as all classes of Proteobacteria. Interrogation of the proteins revealed that there are numerous classes of passenger domains beyond the known proteases, adhesins and esterases. In addition the barrel-domain-a characteristic feature of autotransporters-was found to be composed from seven conserved sequence segments that can be arranged in multiple ways in the tertiary structure of the assembled autotransporter. One of these conserved motifs overlays the targeting information required for autotransporters to reach the outer membrane. Another conserved and diagnostic motif maps to the linker region between the passenger domain and barrel-domain, indicating it as an important feature in the assembly of autotransporters.

## Introduction

Bacteria have an extraordinarily diverse array of protein secretion systems [1]–[4]. As recent studies have uncovered new types of secretion systems, the current Type I-Type VI classification system appears to be approaching a need for re-evaluation. The necessity to rationalize the classification systems is exemplified even in the structurally-simplest system, the Type V secretion system, where new discoveries about structure, function and assembly mechanisms have expanded the classification such that it currently includes the Type Va “autotransporters”, Type Vb “two-partner systems”, Type Vc “trimeric autotransporters”, Type Vd “Patatin-like proteins” [5] and Type Ve “intimins and invasins” (Buchanan S.K., *personal communication*). We sought to derive a broad picture of the diversity within the Type Va group, and provide a bioinformatics framework that could be applied to the analysis of other prospective divisions within the secretion systems superfamily.

The Type Va group of proteins, the autotransporters, are defined by three domains: (i) a signal sequence at the N-terminus that enables targeting of the polypeptide to the inner membrane and through into the periplasm, (ii) the secreted passenger domain which encodes the effector function of each autotransporter, and (iii) the β-barrel “translocation domain”, hereafter referred to as the barrel-domain, consisting of a short α-helical linker segment and 12 β-strands that are assembled into a β-barrel in the outer membrane [6]–[10]. The passenger domains secreted via the autotransporter mechanism mediate virulence through quite distinct biochemical activities: mediating physical adhesion via protein-protein interactions (“adhesins”), proteolytic degradation of select host proteins (“proteases”), lipolytic attack of host cell membranes (“esterases”) [10], [11]-and perhaps other as yet uncharacterized activities as well.

Detailed information is available for a few autotransporters, including crystal structures of passenger domains that suggested some generalizations concerning autotransporter structure and biogenesis. In the well-studied cases, enzymatic domains are sandwiched into a highly β-rich structure, creating a long stalked β-helix that can be up to 100 kDa in size [8], [9], [12]. The passenger domain of extracellular serine protease EspP from *Escherichia coli* (PDB: 3SZE) [13], immunoglobulin IgA1 protease IgAP from *Haemophilus influenzae* (PDB: 3H09) [14], adhesion and penetration protein Hap also from *H. influenzae* (PDB: 3SYJ) [15] and the haemoglobin protease autotransporter Hbp from *E. coli* (PDB: 1WXR) [9], [16] clearly illustrates this. Based on these structures, it has been proposed that the passenger domain folding into a β-helix structure occurs at the surface of the cell, and is ultimately the driving force behind translocation of the passenger domain through the narrow pore of the barrel-domain [17]–[20]. However, with relatively few structures available, and few model proteins tested experimentally, it had been unclear how adequately this model would describe the translocation of autotransporters in general.

Recent experiments have challenged the two aspects of dogma that had explained a view of autotransporter biogenesis. Firstly that passenger domains are composed largely of β-helices, a fold that drives the translocation of the passenger through the β-barrel. The crystal structure of the small autotransporter EstA contains instead an α-helical passenger domain bearing no resemblance to the prototypical β-helix structure [21]. It was uncertain, however, whether EstA was a unique exception to the rule, or simply the first example of many autotransporters with passenger domains that do not fit the β-helix dogma. Secondly, that the barrel-domain of the proteins serves as a translocation channel for the passenger domain to pass through the outer membrane. Biochemical studies with IcsA, IgAP and EspP [22]–[24] indicate that passenger domains adopt at least partially folded conformations in the periplasm before translocation across the outer membrane. The small size of the putative protein-conducting channels of autotransporters, all of which to date appear to be β-barrels of ∼1 nm internal diameter, would impede the transport of passenger domains whether folded or unfolded. Furthermore, cross-linking studies have revealed a role of the β-barrel assembly machinery (BAM complex) in assisting the assembly of autotransporter proteins into the outer membrane [25]–[28]. A recent study showed that a transport and assembly module (the TAM) also contributes to autotransporter secretion [29]. This emphasizes the question of whether the term “autotransporter” is an appropriate description for how these proteins reach the bacterial cell surface, and this class of proteins might instead represent substrates for archetypal protein secretion systems in bacteria [29], [30].

The amassed data from bacterial genome sequencing studies provides a rich resource from which to extract a comprehensive picture of the autotransporters. We sought bioinformatic strategies to interrogate these sequences and address questions that impact on our understanding of autotransporter biogenesis and function: (i) Is the three-part classification system (proteases, adhesins, esterases) that was apparent from the 47 characterized autotransporters, sufficient to characterize all autotransporters? (ii) Does a single type of barrel-domain (i.e. “translocation-domain”) exist-as would be predicted if an autotransporter were an “*auto*nomous *transporter*”, rather than a class of substrate protein – or is there diversity in autotransporter barrel-domains? (iii) Is the small protein EstA a unique exception to the rule that autotransporter have extremely large passenger domains to drive protein translocation across the outer membrane? The answers to these questions provide a means to set the recent experimental work on a few key, model autotransporters in a general context.

We devised a hidden Markov model (HMM) strategy to predict autotransporters from genome sequence, and ultimately detected 1523 protein sequences as putative autotransporters. Cluster-based analysis of the barrel-domains of these proteins shows at least 14 types of β-barrel structures, composed from sequence motifs that define the autotransporter (Type Va) family in general. Conserved motifs define these various types of barrel-domain and the α-linker. Far from being unique, EstA is but one of many autotransporters with very small passenger domains which do not have an extensive β-structure. Conversely, the largest autotransporters detected in this study are ∼5-fold greater in size than the largest autotransporters previously reported. We find a conserved motif, the β-motif, which is common to all autotransporters and often, but not always, found as the final β-strand in the barrel-domain. The β-motif has similar characteristics to the targeting signal that sends eukaryotic β-barrel proteins to the mitochondrial outer membrane [31] and diverse types of β-barrel proteins to the bacterial outer membrane [32]. Taken together, these features define autotransporters as a unified group of substrate proteins, of distinct types and considerable variation, which are transported by the cellular machinery that recognizes them for assembly at the outer membrane.

## Results

### Hidden Markov Models Help Define the Distribution of Autotransporters in Bacteria

**Figure 1:**
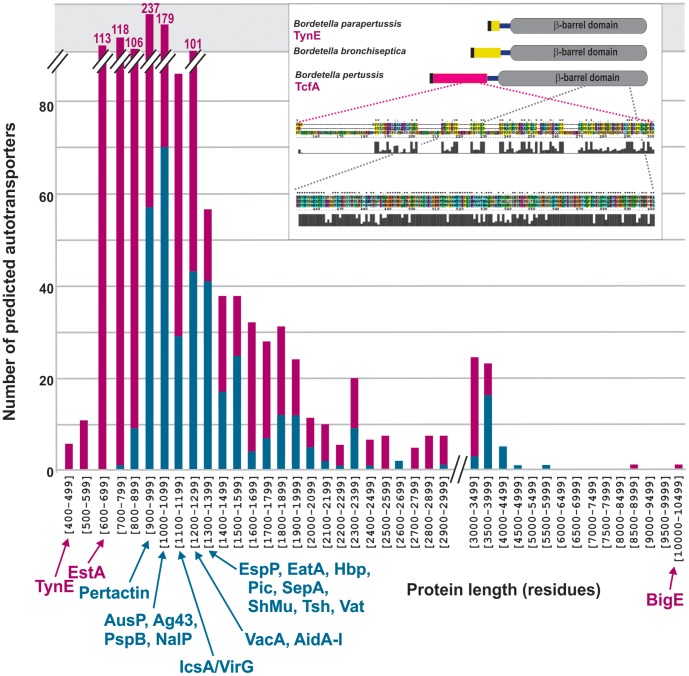
Size distribution of predicted autotransporters. A HMM built to describe the known 47 autotransporters was used to screen bacterial genomes and identified a total of 373 putative autotransporters (using an E-value of 10^−5^ as a cut-off score). These stage 1 results were plotted to the size of the protein sequence (blue bars). A second HMM based on features of the barrel-domains was then used to screen sequences that fell in the range (10^−5^ to 10^−2^) that might be false-negatives in the primary search, and these sequences represented with pink bars. Characterized autotransporters are indicated according to their size either in blue (stringent search criteria) or pink (relaxed search criteria). Details of the largest autotransporter, BigE, are shown in Figure S1. INSET: Representation of the small autotransporters from three species of *Bordetella*: NP_883896.1 [*Bordetella parapertussis* 12822, TynE, 491 residues], NP_889647.1 [*Bordetella bronchiseptica* RB50, 528 residues] and NP_879974.1 [*Bordetella pertussis* Tohama I, TcfA-tracheal colonization factor precursor, 647 residues]. Representative sections of multiple sequence alignment from the passenger domain (colored) and barrel-domain (grey) are shown. The numbering refers to the residues from TcfA, and proline residues (which are prevalent in the first two autotransporters) are colored yellow. The grey histogram plots represent sequence identity (as determined by PsiPred) across all three sequences.

We sought a new strategy based on hidden Markov models (HMMs) to identify autotransporters encoded within genome sequence data. Our aim was to develop a search strategy with ease-of-use and high-sensitivity, to detect autotransporters in diverse bacterial species. At the inception of this study, literature reports had identified 47 proteins in various classes of Proteobacteria where experimental evidence validated them as autotransporters (Table S1). A HMM was built to describe the sequence features of these 47 autotransporters using the entire sequence of each protein. This “AT47-HMM” was used to screen more than a thousand bacterial and archeal genomes from which 373 putative autotransporters were identified with high confidence, using an E-value of 10^−5^ as a cut-off. The E-value 10^−5^ was chosen because within this cut-off, the protein sequences identified (*a*) fall into a size distribution consistent with what is expected from the size of known autotransporters (Figure 1, blue bars) and (*b*) have a predicted barrel-domain that matches to the Pfam “autotransporter” characteristics (pfam03797) as determined by the conserved domain architecture tool CDART [33].

**Figure 2:**
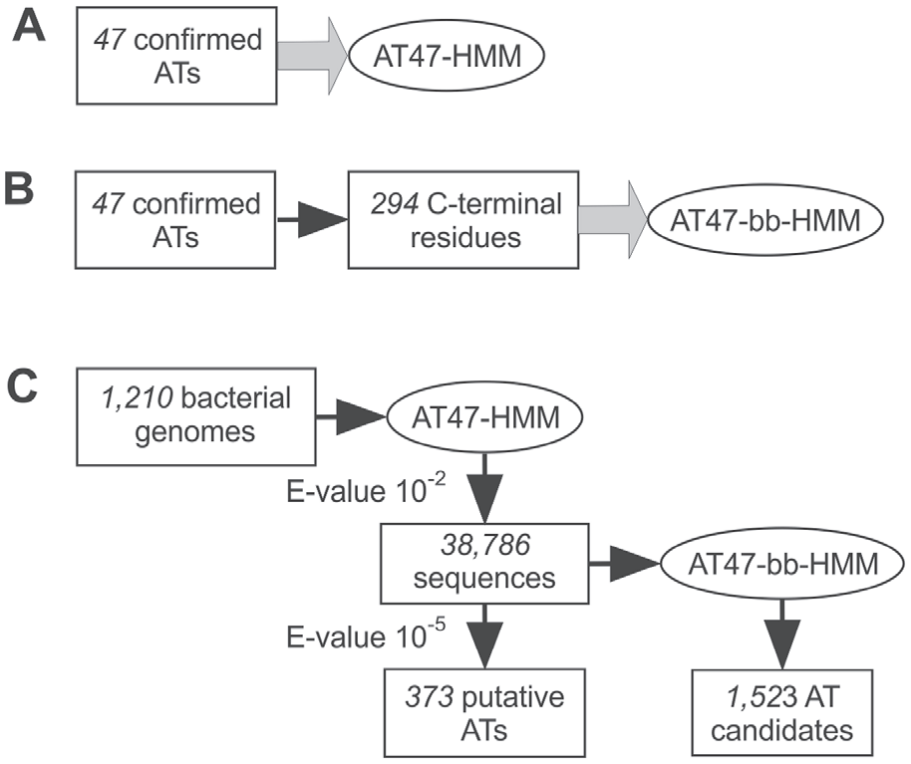
The twin HMM search strategy for autotransporter detection. Panels (A) and (B) depict the building of HMMs: both AT47-HMM and AT47-bb-HMM were initiated from the manually curated list of 47 autotransporters reported in the literature. (A) AT47-HMM was built from full-length autotransporter sequences. (B) AT47-bb-HMM was built from barrel-domains predicted using the secondary structure prediction tool DomPred. (C) Workflow of HMM analysis is shown wherein 1,210 bacterial genomes, comprising a total of 4,264,032 protein sequences, were filtered with AT47-HMM to produce two lists at E-value cutoffs of 10^−2^ and 10^−5^. In the second step, 38,786 sequences collected with the E-value cutoff of 10^−2^ were filtered with AT47-bb-HMM to produce 1,523 AT candidate sequences at the E-value cutoff of 10^−4^. Sequence lists (or sequence databases) are shown as simple rectangles, HMMs are depicted as ovals, simple arrows represent filtering of sequence lists, while thick arrows represent the process of building a HMM from a sequence list.

The atypical autotransporter EstA has a very small, α-helical passenger domain and was not detected within this stringent cut-off. Manual inspection of the search data revealed the EstA sequence to match the AT47-HMM with a score of only 10^−3^. To increase the sensitivity of the search strategy, we sought a second filter to identify sequences that might be false-negatives in the primary search, *i.e.* genuine autotransporters that were missed because they have some atypical sequence characteristics. We therefore set a sufficiently low cut-off to capture this genuine autotransporter (i.e. searched sequences in the E-value range of 10^−5^ to 10^−2^) using a second HMM to detect the presence of autotransporter barrel-domains in the sequences from within this set. To construct this second HMM, barrel-domain sequences were extracted from the 47 known autotransporters (Table S1): these were defined as being present within the C-terminal 294 residues (see Methods). These barrel-domain sequences were used to build a second HMM (named AT47-bb-HMM) expected to recognize the broad features of autotransporter barrel-domains. AT47-bb-HMM was then used to scan the 38,786 sequences detected by AT47-HMM in the E-value range of 10^−5^ to 10^−2^. The twin-HMM strategy, summarized in Figure 2, thereby resulted in a collection of 1523 protein sequences (this included 371 sequences from the initial HMM scan).

Evaluation of the search strategy (see Methods) on test data suggested a very low false-positive detection rate (the estimated rate is 0%), with an acceptable false-negative rate (the estimated rate is 20%). Thus, while the twin-HMM search will underestimate the total number of autotransporters, it would be sensitive enough to detect a wide range of autotransporters from any given genome sequence, while detecting a minimal number of protein sequences which are not autotransporters.

To test this hypothesis we took advantage of a focused study [34] in which an iterative approach using several sequence analysis tools, expert knowledge and functional studies was used to define autotransporter complements of *E. coli* pathotypes. The results from the twin-HMM search are summarized in Table 1 and more details provided in Table S2. The previous study had access to 21 of the 30 genomes now available, and detected 137 autotransporters within this group of 21 genomes. The twin-HMM search detected a total of 143 unique autotransporters in the same genomes. As noted in Table 1, the twin HMM failed to detect 4 proteins that were identified by Wells *et al* as autotransporters: three of these (NP_754777.1, YP_001743716.1, P77286.1) represent sequences which have 100% sequence identity to other sequences in these genomes. As our strategy reports only non-redundant outcomes, these three were not present in the twin-HMM output. The final sequence (UpaH; ACX47353.1) appears to be an example of a false-negative outcome from the twin-HMM search: functional analysis of UpaH concluded that this is an autotransporter, but that the genome sequence for *E. coli* CFT073 in this region was misassembled during genome closure [35], thereby precluding its prediction by our sequence analysis.

**Table 1:** The autotransporters detected in *Escherichia coli.*

Subgroups	Strain	HMM47 search	Target^1^
Avian pathogenic *E. coli* (APEC)	*E. coli* APEC O1	7	7
Enteroaggregative *E. coli* (EAEC)	*E. coli* 55989	10	9
Enterohemorrhagic *E. coli* (EHEC)	*E. coli* O103:H2 str. 12009	8	n/a
	*E. coli* O111:H-str. 11128	4	n/a
	*E. coli* O157:H7 EDL933	10	8
	*E. coli* O157:H7 str. EC4115	6	n/a
	*E. coli* O157:H7 str. Sakai	9	8
	*E. coli* O157:H7 str. TW14359	7	n/a
	*E. coli* O26:H11 str. 11368	9	n/a
Enteropathogenic *E. coli* (EPEC)	*E. coli* O127:H6 str. E2348/69	3	3
	*E. coli* O55:H7 str. CB9615	10	n/a
Enterotoxigenic *E. coli* (ETEC)	*E. coli* E24377A	8	8
Uropathogenic *E. coli* (UPEC)	*E. coli* 536	7	7
	***E. coli*** ** CFT073**	**8**	**10**
	*E. coli* IAI39	5	5
	*E. coli* S88	6	6
	*E. coli* UMN026	11	9
	*E. coli* UTI89	5	5
Commensal strains	*E. coli* ATCC 8739	6	6
	*E. coli* B str. REL606	6	5
	*E. coli* ED1a	7	5
	*E. coli* HS	6	6
	*E. coli* IAI1	5	4
	*E. coli* SE11	7	7
Environmental strains	***E. coli*** ** SMS-3–5**	**8**	**9**
Laboratory Strains	*E. coli* BW2952	5	n/a
	*E. coli* BL21-Gold(DE3)pLysS AG	6	n/a
	*E. coli* K-12 substr. DH10B	5	n/a
	***E. coli*** ** K-12 substr. MG1655**	**4**	**5**
	*E. coli* K-12 substr. W3110	5	5

**Notes:**

1– Analysis by Wells *et al* [34] serves as a benchmark. Cases in which the HMM search apparently underperformed the previous analysis are shown in bold and discussed in main text.

2– n/a indicates genome sequences not previously available.

By way of additional validation for the sensitivity of the twin-HMM strategy, we used the genome of *Citrobacter rodentium* (ICC168) as a bench-mark dataset. *Citrobacter rodentium* is a mouse pathogen used as a model of the human pathogen enteropathogenic *E. coli* (EPEC) because both bacteria cause similar attaching/effacing lesions and share other aspects of pathogenesis [36], [37]. The genome of *C. rodentium* ICC168 has been comprehensively analysed for virulence factors and protein secretion systems based on BLAST search analysis, and 20 autotransporters were reported and catalogued [38]. One of these is a pseudogene resulting from a frame-shift mutation which therefore does not have a corresponding entry in conceptual translations of genome data: the twin-HMM search of the proteins encoded in the genome of *C. rodentium* ICC168 identified the other 19 autotransporters (Table 2).

**Table 2:** The autotransporters detected in *Citrobacter rodentium* (ICC168).

Name	CDS ID^1^	Accession	HMM-47 score	CDART/Pfam Domain match		Homolog (identity)^4^	Passenger type
				passenger	barrel		
EspC	ROD_p1251	YP_003368469.1	0	Peptidase S6	pfam03797^2^	90%	SPATE
Pic	ROD_p1411	YP_003368482.1	0	Peptidase S6	pfam03797	80%	SPATE
Tsh	ROD_41301	YP_003367548.1	9.80E-210	Peptidase S6	pfam03797	84%	SPATE
*Cr*AT4	ROD_03891	YP_003364027.1	6.30E-041	none	pfam03797	–	Recruitment^5^
TibA	ROD_p1121	YP_003368457.1	3.30E-092	PRK09945	pfam03797	78%	AIDA-I-like
Ag43	ROD_49731	YP_003368339.1	2.40E-054	AidA	pfam03797	96%	AIDA-I-like
*Cr*AT7	ROD_00511	YP_003363698.1	2.40E-054	Pertactin	OM channels^3^	–	Recruitment
*Cr*AT8	ROD_11971	YP_003364788.1	9.80E-210	none	pfam03797	–	AIDA-I-like^6^
*Cr*AT9	ROD_16841	YP_003365252.1	6.30E-041	none	pfam03797	–	AIDA-I-like^6^
*Cr*AT10	ROD_11911	YP_003364782.1	3.30E-038	none	pfam03797	–	AIDA-I-like^6^
*Cr*AT11	ROD_16391	YP_003365215.1	3.30E-038	none	pfam03797	–	BrkA
*Cr*AT12	ROD_38761	YP_003367314.1	1.30E-032	MisL	pfam03797	–	Recruitment
*Cr*AT13	ROD_04151	YP_003364053.1	1.30E-032	none	pfam03797	–	AIDA-I-like
*Cr*AT14	ROD_15731	YP_003365154.1	4.10E-032	none	pfam03797	–	Recruitment^5^
*Cr*AT15	ROD_02111	YP_003363855.1	1.80E-031	none	pfam03797	–	Recruitment^5^
*Cr*AT16	ROD_03921	YP_003364030.1	1.40E-021	none	pfam03797	–	Recruitment
*Cr*AT17	ROD_27631	YP_003366294.1	2.10E-021	none	pfam03797	–	BrkA
*Cr*AT18	ROD_03611	YP_003363999.1	1.20E-017	none	pfam03797	–	BrkA
*Cr*AT19	ROD_20811	YP_003365633.1	0.0019	none	OM channels^3^	–	BrkA

**Notes:**

1– As used by Petty et al [38].

2– Pfam03797 is the conserved “autotransporter beta-domain” http://www.ncbi.nlm.nih.gov/Structure/cdd/cdd.shtml.

3– Including a partial match to pfam03797.

4– Closest match by reciprocal BLASTP to characterized protein from strains of *E. coli*.

5– These three proteins share very high sequence similarity.

6– These three proteins share very high sequence similarity.

After this validation, the twin-HMM search strategy was applied to complete genome sequences across 580 species of bacteria and the proteins discovered were plotted onto Figure 1 (pink bars) according to their sequence lengths.

Several of the autotransporters we detected are as small as, and even smaller than, EstA (Figure 1). One of the smallest protein sequences detected is 491 residues long, found in *Bordetella parapertussis* 12822. We refer to this protein as TynE. A homolog of TynE (548 residues in length) is encoded in the genome of in *Bordetella bronchiseptica* RB50. Both of these proteins are predicted to have short, proline-rich passenger-domains and both have signal sequences as predicted by SignalP 3.0. When analyzing only the barrel-domains, these proteins are highly similar (82% sequence identity) to the tracheal colonization factor TcfA found in strains of *Bordetella pertussis.* The passenger domain of tracheal colonization factor is distinct from the shorter proteins (Figure 1, inset), suggesting that a common barrel-domain has been used in combination with distinct passenger domains in species of the genus *Bordetella.*

### Autotransporters are Found in all Five Classes of Proteobacteria, but not in all Species

No autotransporter sequences were detected in viral, archeal or eukaryote genome sequences. In the complete genomes of Proteobacteria currently available, the total number of autotransporters detected by our search is 1344 (Table 3). Autotransporters were found in species of the five classes of the Phylum Proteobacteria (Alpha-, Beta-, Gamma-, Delta-and Epsilon-) but as shown in Table 3, not in all species of Proteobacteria.

**Table 3:** Autotransporter distribution in Phylum Proteobacteria.

Class	Total species	Species withhits	Totalhits	Characterized examples
Alpha-proteobacteria	136	84	309	
Beta-proteobacteria	93	61	212	AusP, IgA-protease, pertactin, NalP
Gamma-proteobacteria	275	151	715	Ag43, AidA-I, EatA, EspP, Hbp, IcsA/VirG, Pic, PspB, SepA,ShMu, TapA, Tsh, Vat
Delta-proteobacteria	39	13	35	
Epsilon-proteobacteria	36	27	73	VacA
**Total**			1344	

**Notes:**

In addition, 93 autotransporters were detected in genomes from other bacterial groups (Fusobacter and Chlamydiales). References for the functionally characterized autotransporters are to be found in Table S1.

In addition to the Proteobacteria, putative autotransporters were also detected in species of Fusobacteria and Chlamydiales. Twenty-four putative autotransporters were previously identified in Fusobacteria using PSI-BLAST approaches [39]. Our study, as detailed below, shows that most of the fusobacterial proteins are highly similar over the entire sequence length to the proteobacterial proteins CapA and CapB, autotransporters that function as adhesins and which are characteristic of the proteobacterium *Campylobacter jejuni* [40]; this strongly suggests that the fusobacterial genes were acquired from a lateral gene transfer event from *Campylobacter* sp. In the Chlamydiales, the polymorphic membrane protein PmpD from *Chlamydia trachomatis* is a type member of the group of 93 proteins detected in the twin-HMM search in species of *Chlamydia* and other Chlamydiales. Previous functional studies on PmpD characterized it as an autotransporter [41], [42].

The twin-HMM search revealed that the number of autotransporters in a given bacterial species ranges from 0 to 19. The highest number was found in *Citrobacter rodentium* (ICC168) which encodes 19 putative autotransporters: only five represent proteins that were known in *E. coli* (corresponding to the *C. rodentium* homologs of EspC, Pic, Tsh, TibA and Ag43): the remaining fourteen are orphan proteins without obvious sequence identity to proteins of known function or characterized passenger domain structures (Table 2).

### Passenger Domains: Functional Super-groups and Domain Evolution

**Figure 3:**
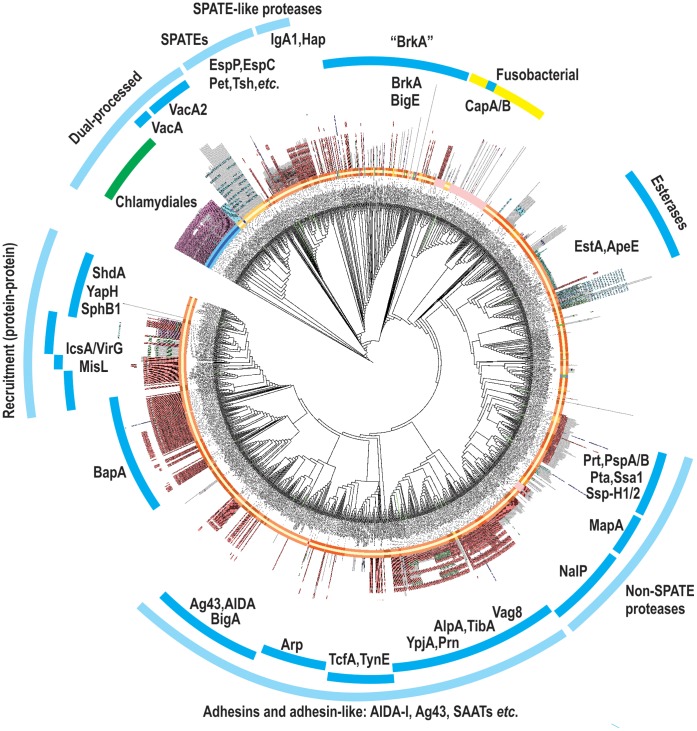
Phylogenetic analysis identifies major families of functional domains. Sequences corresponding to the passenger domain plus α−linker-domain of autotransporters were defined and subject to phylogenetic analysis (Methods). Sub-domain signatures were identified using Pfam analysis of all sequences, and these are represented as radiating coloured symbols. The length of this line is proportional to the number of residues in the passenger-α-linker-domains. Major functional categories are shown (light blue arcs) based on the conservation of Pfam signatures and phylogenetic clustering. Non-proteobacterial sequences are coloured green (Chlamydiales) and yellow (Fusobacteria). Figure S2 provides the tree in a form where the accession numbers of every sequence can be viewed.

Based on their passenger domains, three broad classes of autotransporter are recognized in the literature: esterases, proteases and adhesins [8], [34], [43]–[45]. The large number of autotransporter sequences now available permitted a cluster-based analysis of the passenger domains as a means of a more detailed classification based on sequence similarities. In Figure 3, both the branching pattern of the tree and the identity of the Pfam domains found in each passenger domain suggest six generalizations. Firstly, based on the 1,523 sequences analysed, the esterases represent only a very limited group of autotransporters (Figure 3).

Secondly, that the proteases which have been defined in three groups could be consolidated into two groups: (i) the SPATEs and SPATE-like proteases and (ii) a hydrolase group in which some are proteases. Based on the few proteins studied so far, the relationship between the serine protease autotransporters of the Enterobacteriaceae (SPATEs) and the SPATE-like proteases, which are equivalent autotransporters found in species that are not members of the family Enterobacteriaceae, has been noted previously [44]. Sequence-based clustering distinguished the non-SPATE proteases as a defined group of proteins (Figure 3), but this same group also includes autotransporters with hydrolase activity other than proteolysis: MapA, for example, has an acid phosphatase activity [46]. This may reflect an evolutionary diversification whereby the same overall passenger domain scaffold has evolved to hydrolyze either protein or non-protein substrates.

Thirdly, a functional grouping of AIDA-I-like proteins typified by Ag43, AIDA and TibA, has previously been proposed to represent the self-associating autotransporters (“SAATs”) [47]. The clustering of sequences in Figure 3 lends support to the proposition that these proteins, and many related to them, have an underlying relatedness at the structural level and the AIDA-I-like grouping might therefore be used predicatively: proteins of unknown function like the EPEC str. CB9615 autotransporter YP_003499044.1 and putative relatives in *C. rodentium* (*Cr*AT8, *Cr*AT9 and *Cr*AT10; Table 2) belong to this group and may function as adhesins capable of self-association according to this proposition.

Fourthly, another major grouping of passenger domains evident in Figure 3 includes IcsA/VirG, which activates the host actin regulatory protein N-WASP by a mechanism that involves recruitment of this host factor to the outer membrane of the invading *Shigella* [48]. Grouping together with IcsA/VirG are a range of proteins including the fibronectin-binding protein ShdA [49] and the subtilisin-like protease SphB1 [50]. While the molecular details of the activity of each autotransporter vary, a common generalized function is to maintain a selective protein-protein interface, which has evolved to specifically recruit N-WASP (IcsA/VirG), fibronectin (ShdA) or outer membrane protein substrates (SphB1).

Fifthly, the BrkA autotransporter from *Bordetella pertussis* is required for serum resistance in this pathogen [51]. There are a large number of other proteins with similar passenger domains to BrkA (Figure 3), including BigE, the largest autotransporter detected in the twin-HMM search (Figure S1). Further characterization is required in order to understand the precise biochemical function of the BrkA/BigE-related proteins, but this group accounts for a substantial proportion of autotransporters.

Finally, the autotransporter sequences from Chlamydiales cluster as a group and the phylogenetic analysis supports a relationship to VacA proteins from *Helicobacter pylor*i (Figure 3); a strong similarity can be observed by several structure-based findings. The chlamydial autotransporter PmpD is secreted by a mechanism that requires dual-processing of the passenger domain into parts [42], [52], [53], as is the case for VacA [54]. A recent study using cryo-electron microscopy noted that the oligomeric structure of PmpD is similar to that seen for VacA [42]. The clustering seen in Figure 3 suggests a sequence-based similarity ties the passenger domains of VacA and PmpD together, consistent with the observed similarities in protein architecture in these dual-processed passenger domains.

### Distinguishing Motifs Map to Structurally Unique Features of the Barrel-domain

**Figure 4:**
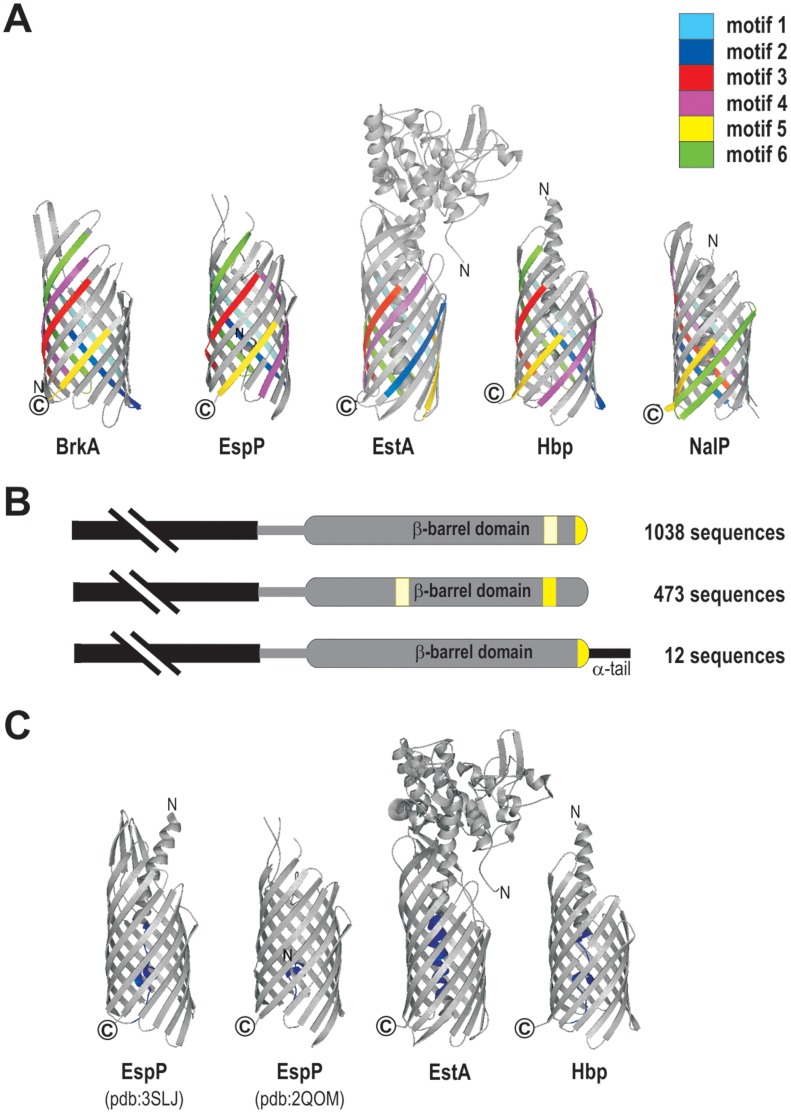
Conserved sequence motifs are not spatially conserved in tertiary structure. (A) MEME was used to identify six common motifs in the barrel-domains of the 47 autotransporter sequences in the starting set (Methods). The six motifs were mapped onto the barrel-domain region of the five autotransporters for which crystal structures have been solved: BrkA, EspP, EstA, Hbp and NalP and are indicated by the respective colours. (B) In 1038 sequences, motif number 5 (the β-motif) was confined to the last β-strand and the final residue in the motif was always a bulky hydrophobic residue. In some of these sequences, a second occurrence, or even a third occurrence of the β-motif was detected in strands other than the terminal strand (paler yellow). In 473 sequences the β-motif is found internally within the barrel-domain. In 12 sequences a short “α-tail” segment succeeds the β-motif of the barrel. Given the topology of bacterial β-barrel proteins, the α-helical tail would presumably be positioned within the periplasmic space. (C) Sequences corresponding to the passenger-and α−linker-domains of autotransporters were defined (Methods) and subject to motif analysis using MEME. A single motif corresponding to the α−linker-domains of 31 of the 47 autotransporter sequences in the starting set (Methods), including EspP, EstA and Hbp was identified and is indicated by blue shading on the crystal structure representations of these proteins. Note, that in the crystallized form of BrkA the corresponding segment of the protein was not visualized.

To describe the common features of the autotransporter barrel-domain, we sought defining sequence motifs. Sequence patterns enriched in the 47 characterized autotransporters (Table S1) were derived using the motif prediction tool MEME, and this revealed six motifs confined to the barrel-domain region of the sequences. These motifs are broadly conserved: when the six motifs were used to scan the 1523 autotransporter sequences, all six motifs were found in all sequences (Methods). The crystal structures of five autotransporter barrel-domains have been solved, and we sought to use the structural information available to determine whether the signature sequence motifs map on to defined structural features in the five barrel domain structures (Figure 4A). Mapping of the six motifs onto the crystal structures of the currently known barrel-domain structures revealed that these did not map in a structurally conserved way: in three-dimensional space, the motifs did not converge to a recognizable feature (Figure 4A).

**Figure 5:**
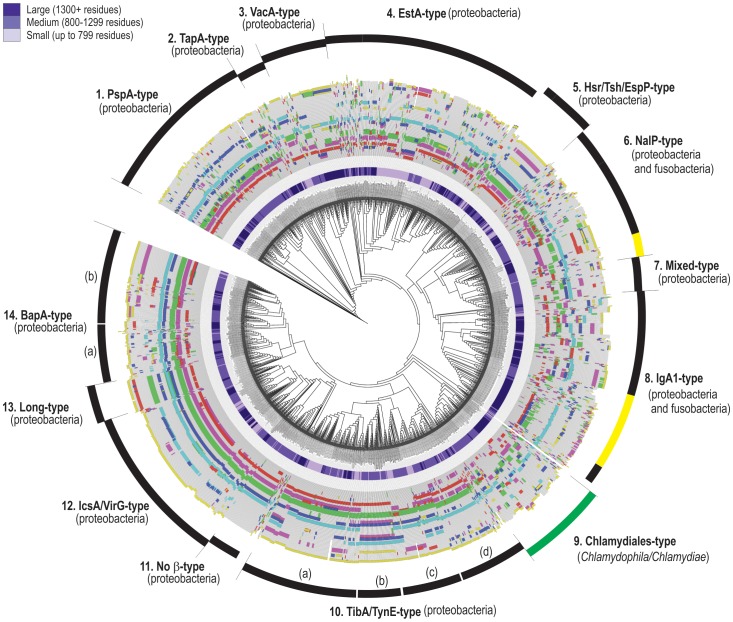
Classification of autotransporter barrel-domains. Sequences corresponding to the barrel-domain of autotransporters were defined and subject to phylogenetic analysis (Methods). Motif analysis of all sequences is represented as radiating coloured stripes, where the colours correspond to those used in Figure 4. The length of the line coloured by these stripes is proportional to the number of residues in the predicted barrel-domain. Fourteen types of barrel-domains were categorized based on conservation of motif placement and phylogenetic clustering. Each type is named for a recognized autotransporter found in that group, for example: Group 1 includes PspA and PspB, Group 4 includes EstA and BigE, Group 6 includes NalP. Group 11 sequences have in common that they have no obvious β-motif, and Group 13 has proteins with longer β-barrel sequences including AIDA-I and Ag43. Each sequence has also been categorized as having a “small”, “medium” or “large” passenger domain, with the purple shading indicating total protein sizes. Figure S3 summarizes the sequence features of the C-terminal (yellow) motifs for each of the 14 groups.

We refer hereafter to motif number 5 as the β-motif. In 1038 sequences (68% of autotransporters), the β-motif was confined to the last β-strand (Figure 4B) and the final residue in the motif was always a bulky hydrophobic residue, often phenylalanine (F) or tryptophan (W) (Figure S3). This terminal aromatic residue has been noted previously as being important for β-barrel assembly into the bacterial outer membrane [32], [55], [56], and an interaction between the last β-strand and the BAM complex has been detected by photo-crosslinking [25]. In some of these sequences, a second occurrence, or even a third occurrence of the β-motif was detected in strands other than the terminal strand (Figure 4B). It appears that its function does not require the β-motif to be in the C-terminal strand, since in 473 sequences the β-motif is found only internally within the barrel-domain (Figure 4B). The structurally defined EstA is one of these proteins: the β-motif is present on the eighth β-strand of the 12-stranded barrel-domain (Figure 4A). Cursory examination of the 473 sequences in which the β-motif is found only internally within the barrel-domain did not reveal these to have other common features, neither with respect to predicted function, nor other sequence similarities (see for example their distribution in Figure5, where the β-motif is coloured yellow).

MEME analysis showed that this β-motif is also found in other groups of outer membrane proteins. Robert *et al* [32] previously analysed a small group of well-studied outer membrane proteins from *E. coli* and *Neisseria meningitidis*, including PhoE, OmpC/OmpF, OmpT, Tsx, FadL, PorA and PorB. In these sequences they identified C-terminal residues that were crucial for binding to the BAM complex, prior to these proteins being assembled into the outer membrane [32]. We extracted the sequences for 19 proteins that had been studied and scanned them with the β-motif using MEME. In fifteen of the nineteen sequences (those of PhoE, OmpC, OmpF, OmpT, Tsx, FadL, Hbp, Pet, PorA, PorB, NspA, Opc, IgA, App and FrpB) there is a statistically significant match to the β-motif that overlaps with the sequence characterized previously by Robert and colleagues [32].

Immediately upstream of the barrel-domain, several autotransporters have been shown to have an α-helical segment that has been observed within the barrel of crystallized autotransporters [21], [57]–[60]. To address whether this is a conserved feature of autotransporters, motif analysis was undertaken using the autotransporter sequences from which the barrel-domain region had been removed (see Methods). These sequences incorporate the passenger domain region as well as any linker segment. Motif analysis (data not shown) showed a complex pattern, but a single conserved motif was detected that maps to the α-helical segment: in the folded protein, this would sit within or immediately outside each barrel-domain and we refer to this conserved sequence segment as the “α-linker motif” (Figure 4C). While the crystal structure of EstA [21] showed it to be atypical in two respects *i.e.* that it had the smallest passenger domain observed in an autotransporter and the passenger domain has an α-helical structure, EstA nonetheless includes a segment corresponding to the α-linker motif (Figure 4C). This sequence conservation suggests that the region corresponding to the α-linker motif is important, independent of the size or structure of the passenger domain. This motif is a novel conserved feature, diagnostic of the autotransporter super-family of proteins.

### A Structure-based Classification of Barrel-domains

The difference in the arrangement of conserved motifs makes a classification of barrel-domain “types” possible. Cluster analysis of the set of 1523 autotransporter sequences suggested the presence of fourteen distinct groups. Wherever possible these were named according to a type of functionally characterized autotransporter: for example Group 1 includes PspA and Ssp-H1 [61] and is named the PspA-type, Group 3 is the VacA-type, etc. Radiating out from each point on the tree shown in Figure 5 is a representation of the barrel-domain drawn to scale, onto which the six motifs are mapped (as characterized in Figure 4A) and accordingly colour-coded. The arrangement and placement of these motifs within a sequence helps to define the fourteen groupings suggested based on our cluster analysis.

The size and structure of the passenger domain does not correlate with the type of barrel-domain. The purple shading in the inner ring of the phylogram in Figure 5 reflects the passenger domain size, and shows clearly that the same type of barrel is used for very small or for very large autotransporters. For example, the autotransporters with the “EstA-type” barrel-domains include both the very large protein BigE, as well as the very small esterases EstA and ApeE.

Given that all autotransporter barrels studied to date consist of 12 β-strands, we were surprised to detect the “long-type” barrel-domains of group 13. These are particularly of interest as this group includes the well-studied adhesins Ag43 and AIDA-I. The placement of the first five motifs is similar in groups 11, 12, 13 and 14, as demonstrated by the colour pattern (Figure 5). However, the sequences in group 13 are distinguished by an additional copy of motif 2, 3 and 6. Structural analysis of these barrels will be necessary to determine whether this represents additional β-strands, or additional structures in the loop regions of these barrels, however we note that in the known structures (Figure 4A) the motifs are found only in transmembrane β-strands.

The passenger domain of BigA is AIDA-I-like and is connected to a Group 6 barrel-domain; this is further evidence that the function of the passenger domain is not related to the type of barrel-domain, with the other members of Group 6 being NalP-type non-SPATE proteases (Figure 5). This mix-and-match feature may reflect an important means by which new autotransporters evolve, but is not consistent with the dogma in which the barrel-domain is specialized for the translocation of its own passenger domain.

## Discussion

The past decade has seen an extraordinary increase in our appreciation of the role of autotransporters in providing crucial bacterial cell functions, particularly with respect to their importance in pathogenesis. Experimental dissection of the structure and function of key, model autotransporters has provided the means to develop and re-assess models for autotransporter biogenesis. Structural analysis of several autotransporters shows that the α-helical linker attached to the passenger domain is embedded within the barrel-domain. However it is becoming clear that the mechanism by which the translocation into the outer membrane occurs is not *auto*nomous-*transport*, but rather performed by several bacterial factors in the periplasm and in the outer membrane that are required to assemble a functionally active autotransporter [10]. Our search strategy revealed more than 1500 putative autotransporter protein sequences, which can be used to define the conserved features of this super-family of proteins, towards a better understanding of how they are assembled and how they have evolved to become so functionally diverse.

### The Diversity and Evolution of Autotransporters

The twin-HMM approach described here provides a means to comprehensively identify autotransporters of different barrel “types” and different passenger domain functionalities. This is a necessary feature of any search strategy, given our finding that passenger domains of given functionality are not fused to a consistent type of barrel-domain. In terms of the evolution of “new” autotransporter functions, there are three important findings from the sequence analysis of this large set of autotransporter sequences.

While not a surprise, there is evidence of both horizontal gene transfer and gene duplication. For example, in *C. rodentium* multiple copies of highly-related autotransporters were evident (Table 2), providing a potential for differential regulation of expression and for the diversification of function through mutation. In regard to horizontal gene transfer as a mechanism of acquiring new autotransporters, we note that various species from the phylum Fusobacteria appear to have adopted proteobacterial autotransporters (*i.e.* proteins related to CapA and CapB) common to species of *Campylobacter*.

Passenger-domain and barrel-domain sequences occur with a “mix-and-match” pattern in our cluster analyses; this is in keeping with a past observation in EHEC EH41 (O113:H21) where a common barrel-domain is attached to EspP, EpeA, and EspI [62], and is further exemplified in the AIDA-I-like proteins that could represent self-associating adhesins. For example, BigA has an AIDA-I-like passenger domain and has a barrel-domain in common with proteins such as the protease NalP. A second example concerning the AIDA-I-like proteins shows that while many of these proteins belong to the group 10a and 10d barrel-domains, AIDA-I (and related proteins such as Ag43) has a distinct, and perhaps larger, barrel-domain.

The highly repetitive β-strand structure of many autotransporter passenger domains [13]-[16], [18], [63], [64] provides for stability and rigidity. However, in evolutionary terms, it also provides for plasticity in accommodating repetitive sequence insertions and other domains [12]. By way of example, the novel protein BigE is 10,429 amino acid residues long and shares a high degree of sequence conservation with a set of smaller proteins from other Beta-proteobacteria. What distinguishes BigE as the largest known autotransporter is an eight-fold replication of the common passenger domain, as well as additional internal sequence repeats with predicted β-strand structure (Figure S1). In addition to extensions in the β-helical framework of the passenger domain, it has previously been observed that some autotransporters are “highly decorated” with small, globular, catalytic domains sandwiched between the repeats of the β-helix [9], [13]–[16]. The evolution of further diversity in passenger domain function is suggested from the structure-based clustering of autotransporters of other functions with the non-SPATE protease group or with the IcsA group. The non-SPATE protease group includes passenger domains with acid phosphatase activity [46], and the group containing IcsA (which binds to the host protein N-WASP; [48]) also includes proteins known to bind to distinct host proteins (*e.g*. Fibronectin; [49]). We suggest that addition of small domains and introduction of variant sequences into the scaffold structure of the passenger domain enabled diversification of protein-substrate interactions needed during the colonization of new niches during evolution.

### The Barrel-domain: Common Sequence Motifs, yet not a Single, Characteristic Barrel

A prediction of the model for *auto*nomous-*transport*, wherein the barrel-domain serves as the translocation pore for passenger domain secretion, is that a functionally conserved type of barrel would be present in all autotransporters. Using the broad diversity of sequences identified in this study, we discovered six enriched sequence motifs in the barrel-domain, and a seventh sequence motif defining the α-linker region, which are together diagnostic of autotransporters. However, the specific arrangement of these motifs defined at least 14 broad “classes” of autotransporter barrel. The striping pattern of six motifs was found to be quite distinct around the assembled barrel, and these positions did not correlate to a type of passenger domain, or to the size of the passenger domain.

One hypothesis is that the conserved sequence features represent segments of the barrel-domain that are bound by factors prior to assembly of the barrel into the outer membrane. This would explain why the order of the motifs within the domain is not important. Recently, several factors representing a targeting and assembly pathway for autotransporters have been identified. For example, SurA interacts with the barrel domain of autotransporters to assist their trafficking to the outer membrane [65]. Structural analysis and peptide-binding experiments have shown that SurA recognizes a tryptic motif corresponding to Aro-x-Aro, where “Aro” is an amino acid residue with an aromatic side-chain and “x” is any residue other than proline [66], [67]. Motifs 2, 6 and the β-motif have such Aro-x-Aro conservation and could represent SurA binding sites. An emerging role for the BAM complex and the recently identified translocation and assembly module (TAM) in autotransporter assembly into the outer membrane might also explain the presence of so many conserved motifs in the barrel-domain [25]–[29], [68]–[70].

In a previous study of diverse beta-barrel proteins, Robert *et al* [32] defined the C-terminal β-strand of several proteins as critical to engage with the BAM complex for assembly into the outer membrane. Our analysis of autotransporters revealed a ubiquitous β-signal, which is often but not always found in the C-terminal β-strand. One example is EstA, where the crystal structure [21] shows that the β-signal occurs in strand 8 rather than strand 12 of the 12-stranded barrel-domain. The C-terminus of EstA does have the generic feature of a conserved, C-terminal aromatic residue, but such residues are conserved by virtue of their role in the “aromatic girdle” that assists β-barrel proteins to register correctly with the membrane boundary [71], [72].

In addition to providing targeting motifs, a further prospect is that one or more motifs in the barrel-domain represent a sequence feature that interacts with the conserved α-linker segment, to help form a “proto-barrel”, a pre-assembly conformation of the barrel. The most critical difference between autotransporters and other β-barrel proteins targeted by SurA to the BAM complex is the need to ensure that the linker peptide will engage with the inner surface of what will ultimately become a folded β-barrel. Mutations within the α-linker prevent correct assembly, such that the passenger-domain of autotransporter Tsh remains in the periplasm while the barrel-domain is inserted into the outer membrane [73]. Recent work on the α-linker region from EspP shows that the region corresponding to the conserved α-linker motif is required to position the α-helical segment within the barrel-domain to promote proteolytic cleavage [74]. This in turn either means that the linker is needed for assembly (that ultimately results in cleavage) or for cleavage *per se*. Finding the α-linker motif in so many autotransporters that are not cleaved casts serious doubt on the suggestion that the primary function of the α-linker is to directly promote the cleavage reaction. While the crystal structure of EstA [21] showed it to be atypical in the structure and size of its passenger domain, EstA is not cleaved during assembly yet nonetheless includes a segment corresponding to the α-linker motif. Altogether, these findings suggest that the region corresponding to the α-linker motif is important, independent of the size or structure or processing requirements of the passenger domain, and we suggest that interactions between the α-linker and conserved segments of the β-barrel are important at an early stage for translocation of diverse autotransporters into and across the outer membrane.

## Methods

### Characterization of Domains for Autotransporter Sequences

Forty-seven protein sequences were selected from the literature on known autotransporters. The list of autotransporters selected is presented in Table S1. Corresponding protein sequences were downloaded from NCBI-Protein database (http://www.ncbi.nlm.nih.gov/protein).

Signal sequence lengths were predicted using SignalP 3.0 (http://www.cbs.dtu.dk/services/SignalP/) [75], and passenger-domain and barrel-domain lengths were determined via secondary structure predictions made by DomPred (http://bioinf.cs.ucl.ac.uk/dompred) [76]. Secondary structure predictions were carried out on the DomPred online server, using default parameters. The first residue of the barrel-domain was defined by the first residue of a β-strand that is preceded by an α-helical and/or coiled-coil region in the expected area were a β-barrel would be expected. The last residue of the barrel-domain was defined as the last residue in the protein sequence. The passenger domain has been defined as the region downstream of the predicted signal sequence and upstream of the predicted barrel-domain. By this definition the passenger domain incorporates the α-helical linker region.

### Hidden Markov Model Analysis

An autotransporter HMM was built with HMMER (http://hmmer.janelia.org/) as previously described [77] using the 47 autotransporter sequences and the HMM build is named AT47-HMM (47 to represent the number of autotransporters in the training dataset).

NCBI-Genome/Bacteria database (ftp://ftp.ncbi.nih.gov/genomes/Bacteria/) was used for HMM analysis. The database consisted of 1331 chromosomal and 927 plasmid files. For each species the taxonomy lineage and total number of proteins encoded were extracted from GenBank files.

The bacterial genome sequence data was scanned using AT47-HMM with HMMER. The scan was carried out with three cut off E-values: 10^−2^, 10^−5^ and 10^−10^, and the protein sequences detected from AT47 HMM were extracted with Yabby (http://code.google.com/p/yabby/). As described in the text, an E-value cut-off of 10^−5^ captured all of the sequences annotated as autotransporters, but did not detect the small autotransporter EstA. Therefore, a secondary search was undertaken after building a HMM of beta-barrel regions of AT47 (henceforth referred to as AT47-bb-HMM). For all hits detected at the E-value cut-off of 10^−2^ in stage 1, sequences were extracted corresponding to the C-terminal 294 amino acid residues (dataset-2), as this was the length of the largest predicted beta-barrel in the sequences in the AT47 group. AT47-bb-HMM was then used to scan dataset-2, using a cut-off value 10^−2^.

A validation for the twin-HMM search strategy was undertaken using the Transporter Classification Database (TCDB), which includes outer membrane proteins and trimeric-autotransporters and other transporters curated from genome data using annotations. TCDB was downloaded from http://www.tcdb.org/and annotated autotransporter sequences were removed. The final dataset consisted of 6067 membrane protein sequences, and was scanned with AT47-HMM and AT47-bb-HMM. No (false-positive) sequences were detected in this dataset.

In order to evaluate the rate of false negatives and false positives to be expected from searches using the twin-HMM search strategy, we made use of protein sequence datasets from PFAM. The PFAM protein family PF03797 (“autotransporter domain”) overlaps with, and is largely equivalent to, the barrel domain of autotransporters. A total of 212 sequences belonging to the PF03797 seed model were retrieved from the UniProt database. Our AT47 models (both AT47HMM and AT47-bb-HMM model) contained 47 sequences manually curated from the primary literature. Comparison with PF03797 showed that 16 sequences were shared (*i.e*. had 100% sequence identity) between the AT47 and PF03797 models. These sequences were removed from the PF03797 set to result in a total of 196 sequences, which we used as the test data set. Application of the twin-HMM procedure to the test data set resulted in 168 hits when the AT47HMM model was applied, and 156 hits when the AT47HMM and AT47-bb-HMM model were applied sequentially. From this we can estimate the false negative rate as 20%. The rate of false positives was estimated by applying the twin-HMM procedure to the test data set consisting of 391 sequences collected from four PFAM models associated with protease activity (PF01478, PF07486, PF06262, and PF08386). We chose protease activity because (a) some autotransporters are proteases, but (b) most proteases are not autotransporters. Application of the HMMs resulted in only two hits when the AT47HMM model was applied, and zero hits when AT47-HMM and AT47-bb-HMM models were applied sequentially. This suggested that the proposed twin-HMM procedure was a highly conservative method for the detection of autotransporters. More specifically, in the genomes analyzed we expect to detect 80% of autotransporters with the rate of false positives close to zero.

### Motif Analysis of Autotransporter Domains

All calculations were carried out locally on a Linux system unless indicated otherwise. Conserved regions were investigated using MEME 4.5.0 (http://meme.sdsc.edu/) [78]. Global alignment of autotransporter sequences used the program needle from the EMBOSS suite. Sequences showing similarity greater than 95% identity were removed to minimize any bias. Motif analysis was carried out on the remaining 39 sequences: we sought conserved regions that were 5 to 200 residues long, present in all 39 barrel-domains, and with the E-value cut-off of 10^−5^. These conserved regions were visualized on the 3D structures of BrkA (PDB: 3QQ2) [79],EspP (PDB: 2QOM) [57], EstA (PDB: 3KVN) [21], Hbp (PDB: 3AEH) [60] and NalP (PDB:1UYN) [59] using PyMOL (http://www.pymol.org). Protein structures were downloaded from RCSB PDB (www.pdb.org) [80].

Analysis of the passenger domains (including the α-linker region) was undertaken by a similar means. Global sequence alignment was carried out on the training set passenger domains using EMBOSS-needle. Sequences showing similarity greater than 95% were removed and then motif analysis was carried out on the remaining 46 sequences. Conserved regions that were 5 to 300 residues long, present in all 46 passenger domains, and had a statistical significance (E-value) of 10^−5^ were reported. Graphical representations (logos) of barrel-domain motifs sequences for 1523 putative autotransporters were generated using WEBLOGO (http://weblogo.berkeley.edu/) [81], [82].

### Clustering of Autotransporter Sequences

Multiple sequence alignment of protein sequences was performed with ClustalW (http://www.clustal.org/) [83]. Phylogenetic tree construction was carried out using PHYLIP (http://evolution.gs.washington.edu/phylip.html) [84]. Phylogenetic trees were displayed and annotated with iTOL-Interactive Tree of Life (http://itol.embl.de/) [85], [86]. The barrel-domain region of 1523 sequences was predicted with AT47-bb-HMM. The barrel-domain in the 1523 sequences were determined using HMMER. The phylogenetic tree shown in Figure 5 was constructed using parsimony, with an outlier sequence Omptin from *E. coli* B088 (ZP_06661096.1). Six conserved regions detected in AT47 barrel-domain region, were scanned for in 1523 barrel-domain regions using HMMER. The passenger domain region was defined as downstream of predicted signal sequence and upstream of the barrel-domain in each of the 1523 sequences. Conserved domains were determined using Pfam-A families (http://pfam.janelia.org/) [87] scanned with HMMER at E-value 0.01. The phylogenetic tree in Figure 3 was constructed using parsimony with pullulanase (PulA) from *Klebsiella pneumoniae* 342 (YP_002240364.1) serving as the outlier.

## Supporting Information

Figure S1 **(A) The barrel-domain of BigE (shaded grey) shares 40% sequence identity (60% sequence similarity) to that of the protein from** ***Ralstonia*** **.** A segment of unknown function from 220–528 (green) is repeated 8 times in BigE. BLAST searches revealed proteins related to the autotransporter from *Ralstonia* in five other species of Beta-Proteobacteria (YP_001354166.1 from *Janthinobacterium* sp. Marseille; YP_005028306.1 from *Dechlorosoma suillum* PS; YP_002355628.1 from *Thauera* sp. MZ1T; YP_004846128.1 from *Pseudogulbenkiania* sp. NH8B; YP_002947691.1 from *Variovorax paradoxus* S110). The black box represents the N-terminal signal sequence of each protein. (B) Distinct regions of the passenger domain of BigE are highlighted: green-the series of domains noted above, yellow-internal repeats, grey-the barrel-domain. The internal repeat sequence highlighted in yellow is plotted as a Sequence Logo: it represents eight repeats of 69 amino acid residues. Hydrophobic residues are coloured black. Hydrophillic residues are coloured green, purple (amines) or red (acidic) according to their side-chain characteristics. The height of the letters indicates how well conserved a residue is in each position of the repeat. PsiPred secondary structure prediction suggests repeated β-strands (each indicated with an arrow) throughout this section of the BigE passenger domain.
(PDF)

Figure S2 **Phylogenetic analysis of functional (passenger) domains.** The active diagram contains all accession information for the 1523 autotransporter sequences. Sub-domain signatures were identified using Pfam analysis of all sequences, and these are represented as radiating coloured symbols. The length of this line is proportional to the number of residues in the passenger:α-linker domains.
(PDF)

Figure S3 **Logos characteristic of the β-signal motif.** In order to best represent the conserved features inherent in motif 5, a sequence Logo was constructed for the motif 5 sequences from each of the 13 (of 14) classes of barrel-domains shown in Figure 5. Some subtle differences are evident between the classes. In each case, the height of the letter representing each amino acid residue is proportional to how well conserved that residue is across the collection of sequences. Amino acid residues are colored according to chemical properties (basic  =  blue; acidic  =  red; hydroxyl  =  green, relatively hydrophobic  =  black).
(PDF)

Table S1 **Forty-seven autotransporters dataset.**
(PDF)

Table S2 **Autotransporter detection in** ***E. coli*** **pathotypes.**
(PDF)
