# Supplementary material for: A Bioinformatic Strategy for the Detection, Classification and Analysis of Bacterial Autotransporters
Source: PLoS One. 2012 Aug 14;7(8):e43245. doi: 10.1371/journal.pone.0043245 (PMC3419190; doi:10.1371/journal.pone.0043245)
Supplement: Figure S1 — (A) The barrel-domain of BigE (shaded grey) shares 40% sequence identity (60% sequence similarity) to that of the protein from Ralstonia . A segment of unknown function from 220–528 (green) is repeated 8 times in BigE. BLAST searches revealed proteins related to the autotransporter from Ralstonia in five other species of Beta-Proteobacteria (YP_001354166.1 from Janthinobacterium sp. Marseille; YP_005028306.1 from Dechlorosoma suillum PS; YP_002355628.1 from Thauera sp. MZ1T; YP_004846128.1 from Pseudogulbenkiania sp. NH8B; YP_002947691.1 from Variovorax paradoxus S110). The black box represents the N-terminal signal sequence of each protein. (B) Distinct regions of the passenger domain of BigE are highlighted: green-the series of domains noted above, yellow-internal repeats, grey-the barrel-domain. The internal repeat sequence highlighted in yellow is plotted as a Sequence Logo: it represents eight repeats of 69 amino acid residues. Hydrophobic residues are coloured black. Hydrophillic residues are coloured green, purple (amines) or red (acidic) according to their side-chain characteristics. The height of the letters indicates how well conserved a residue is in each position of the repeat. PsiPred secondary structure prediction suggests repeated β-strands (each indicated with an arrow) throughout this section of the BigE passenger domain. (PDF) [file pone.0043245.s001.pdf]

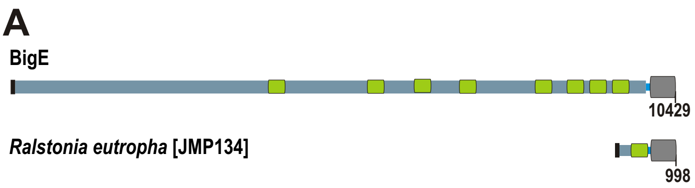

B

BigE

```
MNRIYRLVFNRALGVMQVASEVARNPGGSAVSAGRVPRLRAHQLAVALAATLASGSAFAACTTAGTTVTC
ATGSNTNSLTNSINGVTLNIQTGAELSVPPIVGGSAVNLSGNGITVNNQGTVDPTINGGLSLAASGMVLG
NNTVGGNTMSVNNQAGGAIKGLVNIGSILGFGGQALVVQNASGGVSNIVNAGNVDMSIFGAGLLTTADAA
SIVSYGGAQTTLNNTGTITGRIGFQGSATAGAGNTFLNAGTINGSVNLGSSVAGNTFTAVSGSSVNTAGI
GVAGQVGALAVNLAAAGIVDGGSASNNSLVLQNSATGPGSGTGGAVTTLGWNQYINFQRLTVNSGTWNLQ
GAWAGAGTTTLNDGLVNFNNAGSFGTGLFTANGGAIAASTAGLNLTNAFNLGGNLSLGGTNAFSLGGVLS
GTGGLTVNNTGIVTLGGANLFSGGVNLNAGGLLLGNAGALGSGNLTVGGSASLDTTSGFTLGNNLVINGG
GSLNLLGSNALSLSGSISGAGNVTKNGAGTLTLNGANSLTGAFNLAGGALALGAGGSLSPTGAITLGVGT
SLDLSAATNQTIGSLAGLGGSVNLGSHTLTLGGLGNTSYSGTFAAGTGGLIKLGTGVQTLSGVNNLSGGV
ALNAGGLLLGNAAALGSGTLSVGGNATLDGTTGLALTNAVNLGAGASLNLLGNQALTFNGVIGGAGGLIK
NGAATLTLNGANTFGGGLALNAGSLVLGNGGALGTGALNVGGAVSLDASAGLTVGNGITLGAGGSLDVLG
SNALTLGGVIGGSGSLVKNGASTLTLGGANTFTGGLAVNAGTVSLASGGSLAATGAVSLAGAGSALDISL
GGNQTIGALSGVAGSTLSLGSSTLTFGDATNQTFAGVIGGTGGLIKQGTGVQTLTSASTFSGGVNLAAGG
LVLGNAGALGSGALTVSGNGSLDATAALNLGNAINLGAALTLPGSQNLALAGNVTGTGSLVKNGASTLTL
NGTNTFSGGLSANAGSLLLGNSGALGTGVLGIGGSVSLDGGAPLNLANNVSLGAGAALSLPGSQAITLGG
VVSGAGRLVKNGATTLTLNGANSFGGGLTVNGGGVFLGNAGALGTGALSVGANATLDSSVALNLANDLNL
GAGAALDLLGSQNVSLGGLISGTGSLVKNGTATLALNGANTFSGGVGLNAGLLQLGNAQALGTGQLDVGG
NASLEATTALALGNTIGLAAGANLSVLGSNDLTLAGPVFGAGGLIKDGTARLTLSGANTYNGGTTINAGT
LALGAGGSLAAAGAVNLAVAGATLDISAAGAAQTIGALNGVAGSQLALGAQSLTFGGAGNGSFAGVIGGT
GGLVKTGTGVQTLSGANTFSGGVALNAGGLILGNASALGTGALGIGGTTTLDSTVALNLANAVNFGAGTQ
LTLGGSNDVTLGGVVAGNGSLVKNGAGLLTLNNTNTFGGGLTLNGGGLVVGANGALGTGALAVNASTTLD
AGTAVTLGNAVTLGSGVALTLPGSNALTLSGVVGGNGSLIKNGGTTLTLSGANTYTGGTTINAGTLALGT
GGSLAAVGDVTLGAGAGFDISGAGGNQTIGALNGAGGSTLTLGGNSLTFGTAGNAAFDGVISGGGGLVKV
GAGVQTLSGANTFGGGVTLNAGGLVLGNDAALGAGALTVGGAATLDTTGLATLANNIALNAGLTVIGTNA
LTLNGVLSGTGSLTKNGSATLTLNGANTYTGGTNINAGTLALGAGASLAASGTVNLATGATLDLSAGSGT
QQFGTLVGNGTVNLGANSIEVGGATDGTFSGSIAGTGGLTKVGAGIETLTGANTYTGGTFINAGTLAIGP
GGSLAATGAVTLTAAGTGFDISTTGANQTIGALNGVAGSTVSLGAQTLTLGGVGNSIFDGAISGTGGLVK
NGAGSLTLGGANLFSGGVALNGGALVVGNNAALGSGALTVGSNATLDASTGVSLANNINLASGANLDLLG
SQALTLGGVISGTGGLIKDGAATVTLSGANTYTGGTTINSGTLAIGAGGSLAAAGAVNLAGAGTFDISAG
NQAIGSLAGVAGSTVALGGSTLTLGGTVDSTFSGAISGNGGLVKNGAGVQTLNGASTFSGGVALNAGGLV
VGNNAALGTGAVTVGGAATLDSNTAVTLANNFTLNNALTVLGSNNLTLNGILSGTGSLSKEGAATLTLNG
ANTYTGGTFINAGTLALGAGASLASSGTVNLATGATFDLSAGSGTQQFGTLIGSGTVNLGANTLTIGGPA
NGTFSGSVAGSGGLIKEGSGTQTLTGTNTFTGGTTINAGTLAIGAGGSLAATGAVTLANAGTTFDISAGG
AQAIGSLAGVAGSTVALGGSTLTLNGVGNTTFAGTIGGAGGLVKNGAGIQGLTGVNVFTGGVTLNAGGLL
LGNGGSLGIGALTVGGNASLDGTSALTLANTVQLASGTLTLAGSNALTLNGPISGAGGLLKDGAATVTLG
GASSYTGTTTINSGTLAVAAGGSLSGASTVNLAGAGTLDISAGGNQSIGGLAGVAGSTVALGGATLTTGG
NNASTVFAGVLSGTGGLVQGGTGTLTLSGDNTYTGGTTINGGSTLQIGNGGSTGSVLGDITNNGSLVYNL
GTTATVGAVVSGNGGLTQAGSGTLVLTGNNTYTGGTTINAGGTLQIGNGGATGAIVGDITNNGALVSNVA
GNTTLGGTISGSGGLTQSGSGTLVLTGNNTYTGGTTVNAGGTLQVGNGGATGAITGNVANNGSLVFNVGG
NTTVGGAISGSGGLTQAGSGVLTLVGNNTYTGGTTINTGGTLQVGNGGATGAIAGDITNNGALISNVAGN
TQLGGTISGTGGLTQAGSGSLVLTGTNTYTGGTTINAGGTLQLGNGGATGSIIGDVTNNGTLLSNVAGNT
TLGGTISGSGGLIQAGSGTLTVTGNNTYTGPTTINAGGTLQVGNGGATGAIGGNVTNNGNLVFNVAGNTT
VGGAISGSGGLTQAGSGTLILGGNNTYTGGTTINTGGTLQVGNGGASGAIAGNVSNNGSLVFNVGGNTTV
GGTISGSGGLTQAGTGTVTLTGNNTYTGGTTINAGGTVQVGNGGASGSITGNITNNGGLVINTGGTTTLG
GTISGGGSIVQAGPGGLNLGGNSGGFSGTGQVTGGGLNVTGNIGGQWTIGSGTTLSGTGTIGGTGGGVTV
SSGGVLSPGNGPGNGSGAGNGTGTITVGGNLTLSPGSTLGVDLGASGSDTVQVGGSANVGGSTVQVNTIS
PSTSYQQGQQYTVVNAGGGVSGQFASATSPSAFLTITPVYTANTANLQIDVAQTAAFTTAAKTRNQYNTA
AALDSLPQSGPALGLYNTLLMYDAGTARAAFDQLSGEVHAASRAVLLYDNYLEEGIRQRLGSELPTVRGE
RASAWLAGSGKVFRQDGDGNGDDIRSNRNALMAGVDWQLGEHVVLGAAAGNERLDTRLYDRSSRARLRGN
TFGLYAQGQWNNGFAVGGSVSRGDYRTRTTREVPLLGQTLYSRQDSDVTIAQVEGSWTWTHGRTQLQPYV
QFTRHWVDSDRAVEQGGTAALVLEGGKDTLNVSTVGVRGRWDVGSGERFPAQLTVGLGWQHASGDTDVAS
RNRFAVGGNAFDVYSAVMARNALVSQVGVAVGLGRNSQLSMFVQGQHGDGRRDVGGQINLRVGF
```

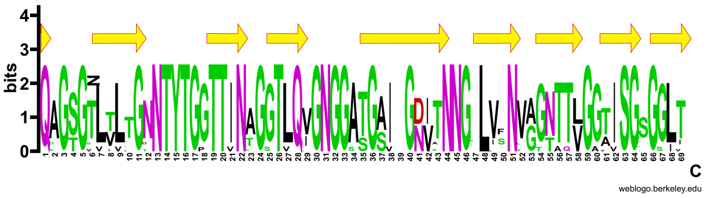

Supplementary Figure S1. (A) The barrel domain of BigE (shaded grey) shares 40% sequence identity (60% sequence similarity) to that of the protein from *Ralstonia*. A segment of unknown function from 220-528 (green) is repeated 8 times in BigE. BLAST searches revealed proteins related to the autotransporter from Ralstonia in five other species of beta-proteobacteria (YP_001354166.1| from *Janthinobacterium* sp. Marseille; YP_005028306.1| from *Dechlorosoma suillum* PS; YP_002355628.1| from *Thauera* sp. MZ1T; YP_004846128.1| from *Pseudogulbenkiania* sp. NH8B; YP_002947691.1| from *Variovorax paradoxus* S110). The black box represents the N-terminal signal sequence of each protein. (B) Distinct regions of the passenger domain of BigE are highlighted: green - the series of domains noted above, yellow - internal repeats, grey - the barrel-domain. The internal repeat sequence highlighted in yellow is plotted as a Sequence Logo: it represents eight repeats of 69 amino acid residues. Hydrophobic residues are colored black. Hydrophillic residues are colored green, purple (amines) or red (acidic) according to their side-chain characteristics. The height of the letters indicates how well conserved a residue is in each position of the repeat. PsiPred secondary structure prediction suggests repeated b-ststrands (each indicated with an arrow).
