# Supplementary figures and images for: A Bioinformatic Strategy for the Detection, Classification and Analysis of Bacterial Autotransporters
Source: PLoS One. 2012 Aug 14;7(8):e43245. doi: 10.1371/journal.pone.0043245 (PMC3419190; doi:10.1371/journal.pone.0043245)

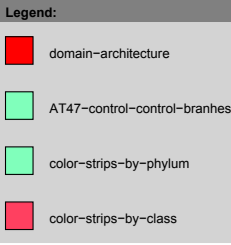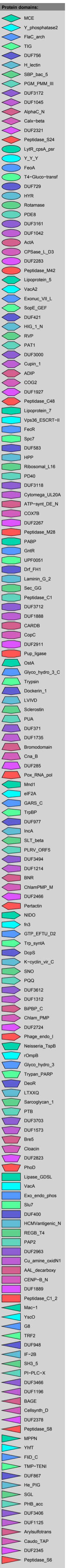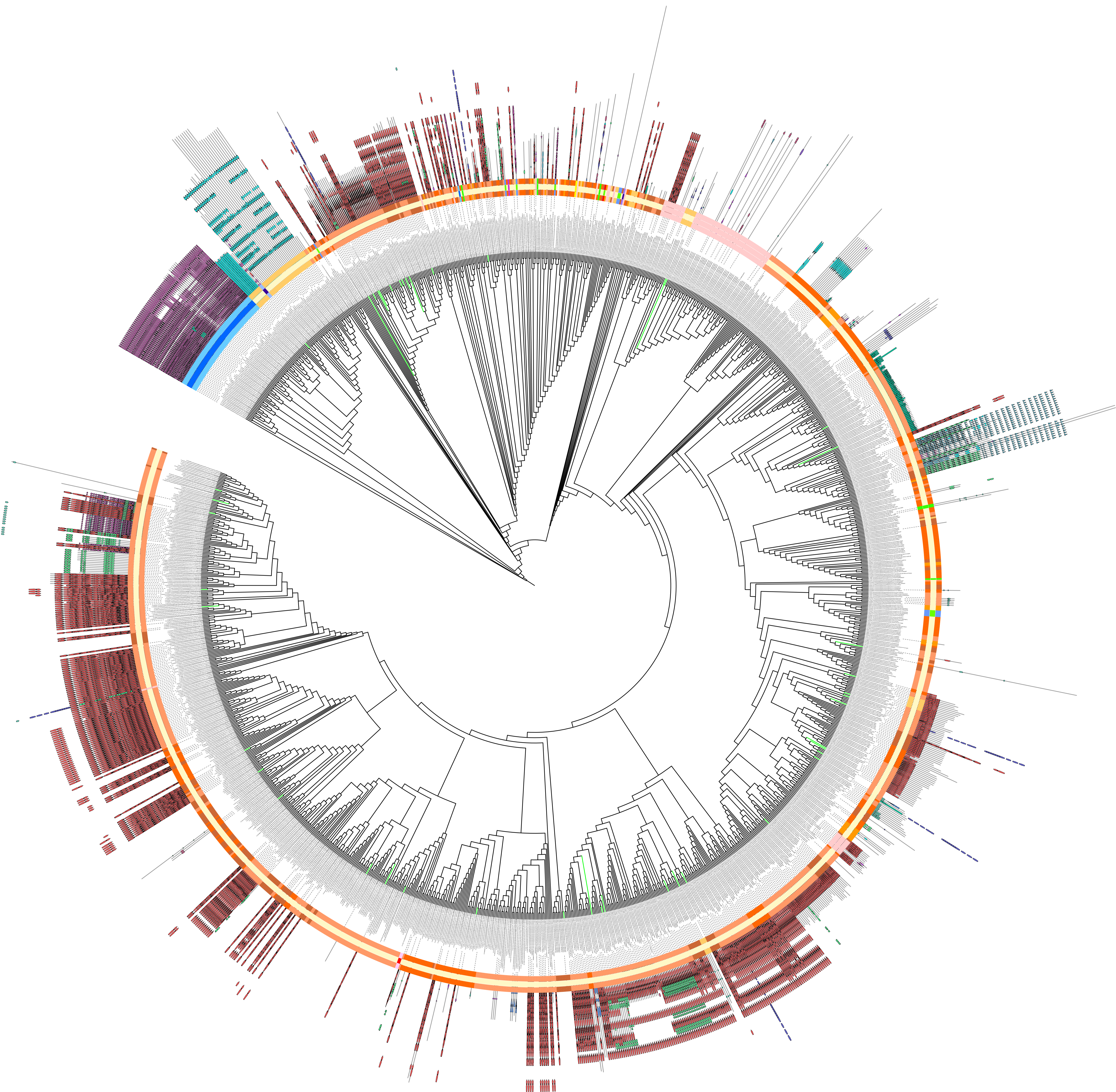

Supplement: Figure S2 — Phylogenetic analysis of functional (passenger) domains. The active diagram contains all accession information for the 1523 autotransporter sequences. Sub-domain signatures were identified using Pfam analysis of all sequences, and these are represented as radiating coloured symbols. The length of this line is proportional to the number of residues in the passenger:α-linker domains. (PDF) [file pone.0043245.s002.pdf]
