# Supplementary material for: A Bioinformatic Strategy for the Detection, Classification and Analysis of Bacterial Autotransporters
Source: PLoS One. 2012 Aug 14;7(8):e43245. doi: 10.1371/journal.pone.0043245 (PMC3419190; doi:10.1371/journal.pone.0043245)
Supplement: Figure S3 — Logos characteristic of the β-signal motif. In order to best represent the conserved features inherent in motif 5, a sequence Logo was constructed for the motif 5 sequences from each of the 13 (of 14) classes of barrel-domains shown in Figure 5. Some subtle differences are evident between the classes. In each case, the height of the letter representing each amino acid residue is proportional to how well conserved that residue is across the collection of sequences. Amino acid residues are colored according to chemical properties (basic = blue; acidic = red; hydroxyl = green, relatively hydrophobic = black). (PDF) [file pone.0043245.s003.pdf]

Supplementary Figure S3. Logos characteristic of the β-signal motif. In order to best represent the conserved features inherent in motif 5, a sequence Logo was constructed for the motif 5 sequences from each of the 13 (of 14) classes of barrel-domains shown in Figure 5. Some subtle differences are evident between the classes, but the general motif can be written with single letter code for the amino acid residues as: G/A-x-x-G-x-R/G-Y/W-x-F (where "x" represents a non-conserved residue). In each case, the height of the letter representing each amino acid residue is proportional to how well conserved that residue is across the collection of sequences. Amino acid residues are colored according to chemical properties (basic = blue; acidic = red; hydroxyl = green, relatively hydrophobic = black).

AT beta-barrel subgroup	Motif Logo
1. PspA-type	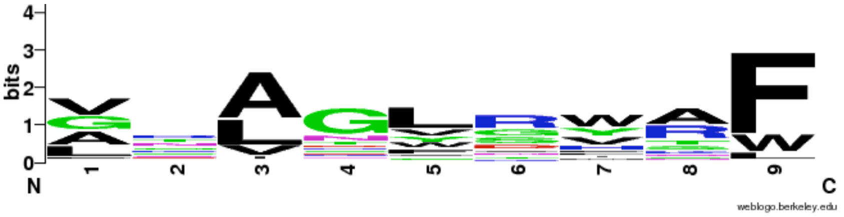
2. TapA-type	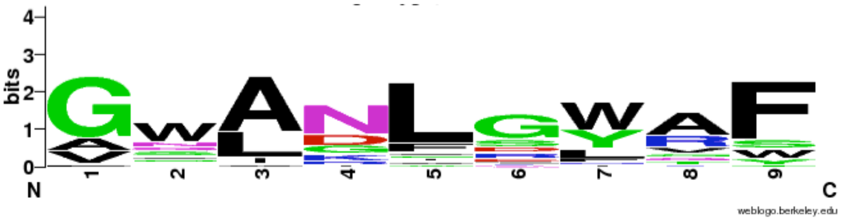
3. VacA-type	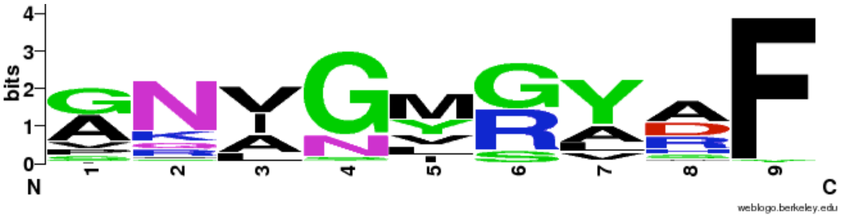
4. EstA-type	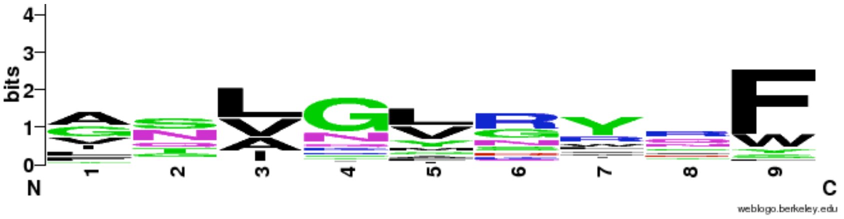

5.Hsr/Tsh/EspP-type	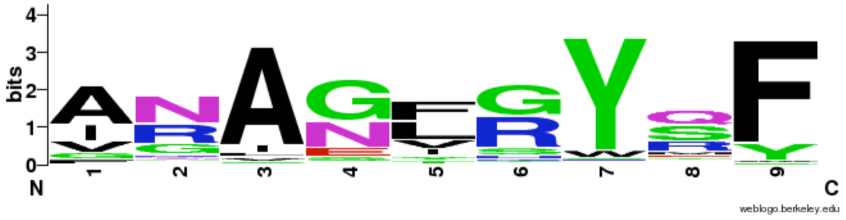
6. NalP-type	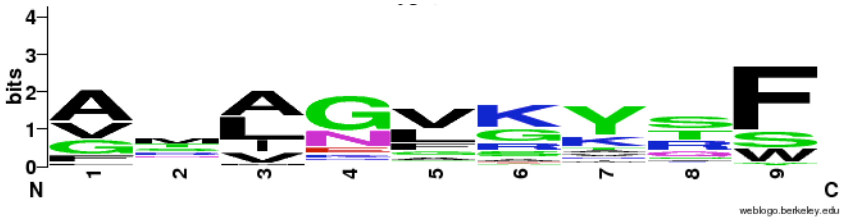
7. Mixed-type	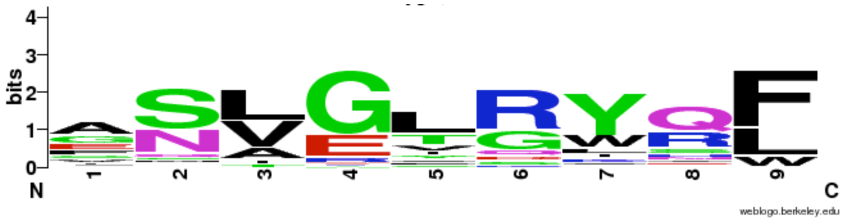
8. IgA1-type	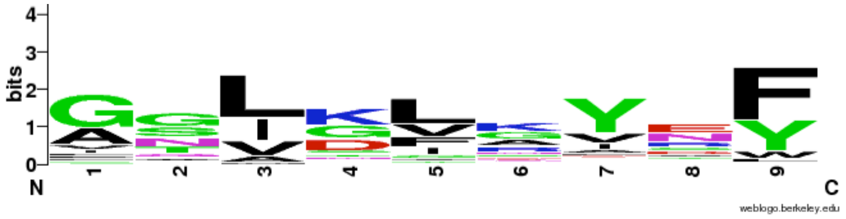
9. Chlamydiales-type	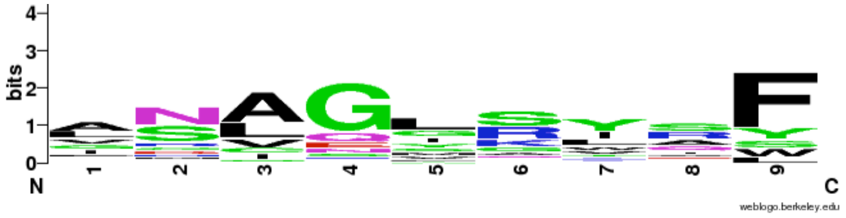
10. TibA/TynE-type	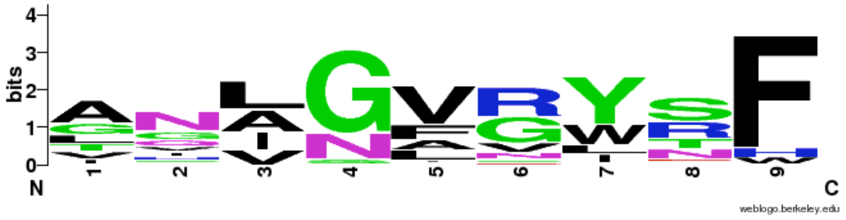

12. IcsA/VirG-type	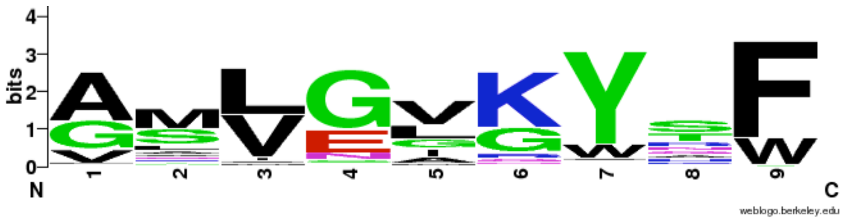
13. Long-type	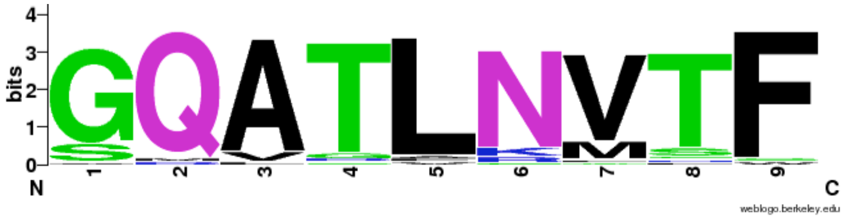
14. BapA-type	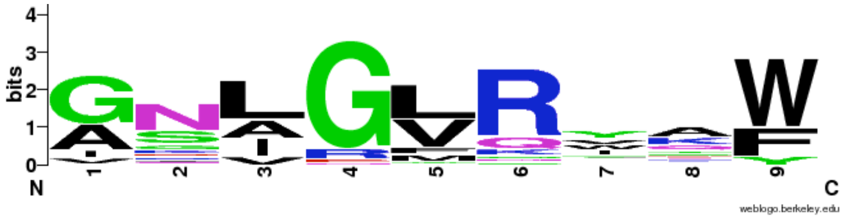
