# Supplementary material for: A Bioinformatic Strategy for the Detection, Classification and Analysis of Bacterial Autotransporters
Source: PLoS One. 2012 Aug 14;7(8):e43245. doi: 10.1371/journal.pone.0043245 (PMC3419190; doi:10.1371/journal.pone.0043245)
Supplement: Table S1 — Forty-seven autotransporters dataset. (PDF) [file pone.0043245.s004.pdf]

Supplementary Table S1. Autotransporter training dataset (47 sequences).

AT	Description	Class	Organism	Function	Pfam03797	NCBI	Length	Reference
AoaA	uncharacterized protein	Alphaproteobacteria	*Azorhizobium caulinodans*		no	BAF88633.1	3766	Suzuki et al 2008
Arp	acidic repeat protein	Alphaproteobacteria	*Bartonella henselae*		no	ABS57467.1	1441	Litwin et al 2007
BapA	putative autotransporter	Betaproteobacteria	*Bordetella pertussis*		yes	CAE42500.1	903	Cummings et al. 2004
BrkA		Betaproteobacteria	*Bordetella pertussis*	Serum resistance	yes	AAA51646.1	1010	Henderson and Nataro , 2001
IgA1	IgA-specific serine endopeptidase autotransporter	Betaproteobacteria	*Neisseria gonorrhoeae*	Protease	yes	P09790.1	1532	Henderson and Nataro , 2001
NalP (AspA)		Betaproteobacteria	*Neisseria meningitidis*		yes	AAN71715.1	1083	Ooment et al 2004
Prn	pertactin	Betaproteobacteria	*Bordetella*	Adhesin	yes	NP_879839.1	910	Henderson and Nataro , 2001
PrtS		Betaproteobacteria	*Bordetella parapertussis*	protease	yes	CAE40231.1	1300	Oliver et al 2003
SphB1		Betaproteobacteria	*Bordetella pertussis*		yes	NP_879378.1	2240	Henderson et al 2004
TcfA	tracheal colonization factor	Betaproteobacteria	*Bordetella pertussis*	Adhesin	yes	ADA85103.1	631	Henderson and Nataro , 2001
Vag8		Betaproteobacteria	*Bordetella pertussis*		yes	AAC31247.1	915	Henderson and Nataro , 2001
CapA	putative lipoprotein	Epsilonproteobacteria	*Campylobacter jejuni*		no	CAL34774.1	1144	Ashgar et al 2006
CapB	putative lipoprotein	Epsilonproteobacteria	*Campylobacter*		no	CAL35773.1	1120	Ashgar et al 2006
Hsr	major ring-forming surface antigen protein Hsr	Epsilonproteobacteria	*Helicobacter mustelae*		yes	YP_003516849.1	1506	Henderson and Nataro , 2001
VacA		Epsilonproteobacteria	*Helicobacter pylori*	Toxin	yes	ADP02391.1	1291	Henderson and Nataro , 2001, Fischer et al. 2001
Ag43	Antigen 43	Gammaproteobacteria	*Escherichia coli*	Biofilm formation/adhesin	yes	P39180.3	1039	Henderson and Nataro , 2001
AIDA-I		Gammaproteobacteria	*Escherichia coli*	Adhesin	yes	CAA46156.1	1286	Henderson and Nataro , 2001
AlpA(ORF_f949)		Gammaproteobacteria	*Escherichia coli*		yes	AAA79815.1	949	Henderson et al 2004
ApeE	outer membrane esterase	Gammaproteobacteria	*Salmonella enterica serovar Typhimurium*	esterase	yes	AAC38796.1	656	Henderson and Nataro , 2001,Henderson et al 2004
BigA		Gammaproteobacteria	*Salmonella enterica* subsp. enterica serovar Montevideo		yes	EGA51972.1	1951	Tukel et al 2007
EspC		Gammaproteobacteria	*Escherichia coli*	enterotoxin	yes	AAC44731.1	1306	Drago-Serrano et al. 2006
EspP	Serine protease espP	Gammaproteobacteria	*Escherichia coli*	Proteolytic toxin	yes	O32591.1	1300	Barnard et al. 2007
EstA	Esterase	Gammaproteobacteria	*Pseudomonas aeruginosa*	esterase	yes	O33407.1	646	Bert van den Berg 2010
Hap	Adhesion and penetration protein autotransporter	Gammaproteobacteria	*Haemophilus influenzae*	adhesin/protease	yes	P44596.2	1409	Henderson and Nataro , 2001
Hbp	haemoglobin protease	Gammaproteobacteria	*Escherichia coli*	haemoglobin protease	yes	CAA11507.1	1377	Otto et al. 2005
IcsA (VirG)	Outer membrane protein IcsA autotransporter	Gammaproteobacteria	*Shigella flexneri*	Mediator of intracellular motility	yes	Q7BCK4.1	1102	Henderson and Nataro , 2001
MapA	acid phosphatase autotransporter	Gammaproteobacteria	*Moraxella catarrhalis*		no	ABP88873.1	940	Hoopman et al 2008
MisL		Gammaproteobacteria	*Salmonella enterica s*ubsp. Enterica serovar Typhimurium		yes	AAD16954.1	955	Tukel et al 2007
Pet		Gammaproteobacteria	*Escherichia coli*	Proteolytic toxin	yes	AAC26634.1	1295	Henderson and Nataro , 2001
Pic	Pic serine protease	Gammaproteobacteria	*Escherichia coli*	Mucinase	yes	AAD23953.1	1372	Henderson and Nataro , 2001
PrtT		Gammaproteobacteria	*Pectobacterium atrosepticum* SCRI1043	protease	no	YP_049087.1	1036	Henderson and Nataro , 2001
PspA		Gammaproteobacteria	*Pseudomonas fluorescens*	protease	yes	BAA36466.1	985	Henderson and Nataro , 2001
PspB	serine protease	Gammaproteobacteria	*Pseudomonas fluorescens*	protease	yes	BAA36467.1	1036	Henderson and Nataro , 2001
Pta	serine protease	Gammaproteobacteria	*Proteus mirabilis*	Serine protease	no	CAR44626.1	1084	Alamuri el al 2009
Sat	Serine protease sat autotransporter	Gammaproteobacteria	*Escherichia coli*	Proteolytic toxin	yes	Q8FDW4.2	1295	Parham et al. 2004
SepA		Gammaproteobacteria	*Shigella flexneri*	protease/inflammation/invasion	yes	CAA88252.1	1366	Henderson and Nataro , 2001
ShdA		Gammaproteobacteria	*Salmonella enterica* subsp. Enterica serovar Typhimurium		yes	AAD25110.2	2035	Tukel et al 2007
SigA		Gammaproteobacteria	*Shigella flexneri*	Proteolytic toxin	yes	AAF67320.1	1285	Henderson and Nataro , 2001
Ssa1	Serotype-specific antigen 1	Gammaproteobacteria	*Pasteurella haemolytica*	protease	yes	P31631.1	932	Henderson and Nataro , 2001
Ssp-H1		Gammaproteobacteria	*Serratia marcesens*	protease	yes	BAA33455.1	1036	Henderson and Nataro , 2001
Ssp-H2		Gammaproteobacteria	*Serratia marcesens*	protease	yes	BAA11383.1	1034	Henderson and Nataro , 2001
TapA		Gammaproteobacteria	*Acidithiobacillus ferrooxidans* ATCC 23270		yes	ACK80564.1	991	Dautin and Bernstein, 2007
TibA		Gammaproteobacteria	*Escherichia coli*	adhesin	yes	AAD41751.1	989	Dautin and Bernstein, 2007
Tsh		Gammaproteobacteria	*Escherichia coli*	Hemagglutinin/hemoglobin binding	yes	AAA24698.1	1377	Henderson and Nataro , 2001
Vat	vacuolating autotransporter toxin	Gammaproteobacteria	*Escherichia coli*		yes	AAO21903.1	1377	Parham et al. 2004
YapH		Gammaproteobacteria	*Yersinia pestis*		yes	CAC14227.1	3705	Dautin and Bernstein, 2007
YpjA(EhaD)		Gammaproteobacteria	*Escherichia coli*		yes	AP_003226.1	1526	Wells et al. 2010
