# Supplementary material for: A Bioinformatic Strategy for the Detection, Classification and Analysis of Bacterial Autotransporters
Source: PLoS One. 2012 Aug 14;7(8):e43245. doi: 10.1371/journal.pone.0043245 (PMC3419190; doi:10.1371/journal.pone.0043245)
Supplement: Table S2 — Autotransporter detection in E. coli pathotypes. (PDF) [file pone.0043245.s005.pdf]

Supplementary Table S2. Escherichia coli hits.

Escherichia coli strain	Total Hits	Strain Accession	Total Proteins	Total Chromosome/Plasmid Hits	Protein Accession	Protein Description	AT47-HMM score	AT47bb-HMM score
Escherichia coli 536	7	NC_008253	4619	7	YP_668278.1	SepA	0.00E+000	2.90E-078
					YP_668265.1	putative autotransporter	1.10E-087	1.10E-057
					YP_670893.1	putative autotransporter	2.60E-050	8.80E-057
					YP_668312.1	putative autotransporter	2.70E-004	1.30E-047
					YP_668363.1	puative autotransporter/pertactin	1.10E-004	4.80E-045
					YP_670171.1	adhesin	3.40E-008	8.50E-042
					YP_669320.1	putative autotransporter/adhesin	1.80E-004	4.30E-023
Escherichia coli 55989	10	NC_011748	4759	10	YP_002404249.1	Serine protease pic precursor (ShMu)	0.00E+000	7.90E-088
					YP_002405532.1	Serine protease pic precursor (ShMu)	0.00E+000	7.90E-088
					YP_002405547.1	Serine protease pet precursor (Plasmid-encoded toxin pet)	0.00E+000	1.10E-086
					YP_002401441.1	putative beta-barrel outer membrane protein, similar to AidA-I adhesin-like protein	1.90E-072	6.60E-082
					YP_002402715.1	putative lipoprotein	6.20E-049	7.60E-074
					YP_002402351.1	putative Outer membrane autotransporter barrel, putative pectin lyase fold	1.40E-004	1.60E-064
					YP_002404320.1	antigen 43 (Ag43) phase-variable biofilm formation autotransporter; CP4-44 prophage	2.10E-087	1.10E-056
					YP_002401499.1	putative flagellin structural protein; putative exported protein	1.40E-004	6.00E-045
					YP_002403506.1	adhesin	6.20E-005	5.70E-041
					YP_002402383.1	putative adhesin; putative autotransporter	9.30E-005	7.00E-031
Escherichia coli APEC O1	7	NC_008563	4428	5	YP_851472.1	vacuolating autotransporter	0.00E+000	2.90E-078
					YP_851500.1	hypothetical protein APECO1_1684	2.60E-004	1.30E-047
					YP_851550.1	putative structural protein	1.10E-004	4.80E-045
					YP_853340.1	adhesin	6.00E-010	2.60E-041
					YP_852571.1	EntS/YbdA MFS transporter	1.60E-004	9.10E-023
		NC_009837	199	2	YP_001481228.1	Tsh	0.00E+000	3.60E-078
					YP_001481251.1	hypothetical protein APECO1_O1CoBM96	4.40E-015	1.00E-060
Escherichia coli ATCC 8739	6	NC_010468	4199	6	YP_001724046.1	outer membrane autotransporter	6.40E-168	8.20E-082
					YP_001725115.1	outer membrane autotransporter	1.10E-049	7.60E-074
					YP_001724448.1	outer membrane autotransporter	1.30E-006	2.50E-059
					YP_001726207.1	outer membrane autotransporter	1.00E-004	6.00E-045
					YP_001724408.1	adhesin	6.60E-005	3.30E-041
					YP_001725385.1	outer membrane autotransporter	9.00E-005	3.10E-030
Escherichia coli B str. REL606	6	NC_012967	4205	6	YP_003046708.1	hypothetical protein ECB_03531	3.50E-019	2.10E-063
					YP_003045312.1	putative autotransporter outer membrane protein	1.00E-004	2.50E-059
					YP_003045987.1	antigen 43 (Ag43) phase-variable biofilm formation autotransporter	3.50E-056	3.60E-057
					YP_003043548.1	hypothetical protein ECB_00321	1.60E-004	4.80E-045
					YP_003045352.1	adhesin	6.20E-005	1.50E-040
					YP_003044392.1	putative adhesin	8.60E-005	5.40E-031
Escherichia coli BL21-Gold(DE3)pLysS AG	6	NC_012947	4228	6	YP_003036351.1	outer membrane autotransporter barrel domain protein	2.80E-003	7.60E-074
					YP_003034319.1	outer membrane autotransporter barrel domain protein	3.50E-019	2.10E-063
					YP_003035715.1	Pertactin	5.00E-005	2.50E-059
					YP_003037476.1	outer membrane autotransporter barrel domain protein	1.60E-004	4.80E-045
					YP_003035675.1	adhesin	6.20E-005	1.50E-040
					YP_003036631.1	outer membrane autotransporter barrel domain protein	8.60E-005	5.40E-031
Escherichia coli BW2952	5	NC_012759	4084	5	YP_002927598.1	adhesin-like autotransporter	1.40E-167	8.20E-082
					YP_002926525.1	hypothetical protein BWG_1328	6.30E-003	7.60E-074

					YP_002926987.1	CP4-44 prophage; antigen 43 (Ag43) phase-variable biofilm formation autotransporter	4.70E-115	5.20E-061
					YP_002927202.1	adhesin	6.10E-005	5.90E-041
					YP_002926229.1	putative adhesin	9.20E-005	5.40E-031
Escherichia coli CFT073	8	NC_004431	5338	8	NP_752289.1	Pic serine protease precursor	0.00E+000	1.80E-087
					NP_755494.1	Aecreted auto transpoter toxin	0.00E+000	1.20E-085
					NP_752330.1	haemoglobin protease	0.00E+000	2.90E-078
					NP_755530.1	antigen 43 precursor	2.10E-087	3.40E-057
					NP_753187.1	antigen 43 precursor	2.00E-086	1.50E-054
					NP_752363.1	hypothetical protein c0426	2.40E-004	1.30E-047
					NP_752412.1	putative structural protein	1.10E-004	4.80E-045
					NP_754661.1	adhesin	7.00E-012	1.90E-043
Escherichia coli E24377A	8	NC_009790	69	1	YP_001451588.1	secreted serine peptidase EatA	0.00E+000	7.40E-079
		NC_009801	4749	7	YP_001461485.1	putative outer membrane autotransporter	2.40E-069	6.60E-082
					YP_001462797.1	outer membrane autotransporter domain-containing protein	3.50E-050	7.60E-074
					YP_001464349.1	antigen 43	3.30E-057	3.30E-057
					YP_001465820.1	antigen 43	3.10E-056	2.30E-055
					YP_001461547.1	outer membrane autotransporter	2.00E-004	6.00E-045
					YP_001463580.1	adhesin	5.70E-005	1.50E-042
					YP_001462453.1	putative outer membrane autotransporter	8.50E-005	1.70E-029
Escherichia coli ED1a	7	NC_011745	4915	7	YP_002397647.1	peptidase S6, IgA endopeptidase from phage origin	2.10E-236	2.10E-086
					YP_002398404.1	serine protease autotransporter	1.40E-237	2.10E-086
					YP_002398019.1	peptidase S6, IgA endopeptidase from phage origin	2.10E-236	2.10E-086
					YP_002400911.1	antigen 43 (Ag43) phase-variable biofilm formation autotransporter; CP4-44 prophage	1.90E-083	3.10E-053
					YP_002396404.1	putative adhesin	7.30E-004	1.20E-047
					YP_002398608.1	adhesin	3.40E-010	2.70E-041
					YP_002397556.1	putative cell adhesion autotransported outer membrane protein	1.40E-004	1.90E-021
Escherichia coli HS	6	NC_009800	4378	6	YP_001457145.1	putative outer membrane autotransporter	3.20E-070	3.50E-081
					YP_001458990.1	putative autotransporter, IS5K-containing	5.00E-005	2.50E-059
					YP_001457134.1	putative outer membrane autotransporter	2.70E-004	8.50E-048
					YP_001459034.1	adhesin	6.30E-005	5.90E-041
					YP_001458024.1	putative outer membrane autotransporter	8.70E-005	5.40E-031
					YP_001458201.1	autotransporter (AT) family porin	9.40E-005	1.40E-026
Escherichia coli IAI1	5	NC_011741	4351	5	YP_002385790.1	putative beta-barrel outer membrane protein, similar to AidA-I adhesin-like protein	7.60E-070	6.60E-082
					YP_002386957.1	putative lipoprotein	1.00E-047	2.80E-074
					YP_002386639.1	putative Outer membrane autotransporter barrel, putative pectin lyase fold	1.40E-004	4.00E-065
					YP_002385846.1	Putative flagellin structural protein ; putative exported protein	1.30E-004	6.00E-045
					YP_002387709.1	adhesin	5.60E-005	1.60E-043
Escherichia coli IAI39	5	NC_011750	4730	5	YP_002407881.1	putative outer membrane autotransporter barrel, putative pectin lyase fold	3.50E-005	4.80E-065
					YP_002408290.1	putative autotransporter outer membrane protein; type V secretion	8.30E-005	1.10E-061
					YP_002406352.1	putative flagellin-like structural protein	1.10E-004	5.20E-045
					YP_002408332.1	adhesin	8.60E-007	1.30E-041
					YP_002407707.1	putative cell adhesion autotransported outer membrane protein	1.90E-004	1.70E-027
Escherichia coli O103:H2 str. 12009	8	NC_013353	5054	8	YP_003223650.1	secreted autotransporter serine protease	4.80E-286	3.00E-085

					YP_003220297.1	AidA-I adhesin-like protein	1.30E-072	7.20E-082
					YP_003221593.1	putative autotransporter protein	6.50E-052	2.80E-074
					YP_003221241.1	AidA-I adhesin-like protein	1.40E-004	1.60E-066
					YP_003224716.1	putative AidA-I adhesin-like protein	2.00E-054	5.50E-058
					YP_003220352.1	putative flagellin structural protein	1.40E-004	6.00E-045
					YP_003222607.1	adhesin YfaL	5.80E-005	1.60E-043
					YP_003221272.1	putative adhesin	3.80E-004	2.20E-007
Escherichia coli O111:H- str. 11128	4	NC_013364	4972	3	YP_003232885.1	AidA-I adhesin-like protein	2.20E-070	5.40E-082
					YP_003235365.1	adhesin YfaL	3.20E-006	1.60E-043
					YP_003234009.1	putative adhesin	8.60E-005	1.70E-029
		NC_013366	72	1	YP_003237769.1	predicted serine protease EspP	8.50E-275	6.60E-086
Escherichia coli O127:H6 str. E2348/69	3	NC_011601	4552	3	YP_002330403.1	extracellular serine protease EspC	0.00E+000	4.50E-090
					YP_002330202.1	AidA autotransporter-like protein	1.40E-032	1.20E-072
					YP_002329881.1	adhesin	2.40E-009	3.10E-043
Escherichia coli O157:H7 str. EC4115	6	NC_011350	108	1	YP_002268480.1	immunoglobulin A1 protease domain protein	0.00E+000	6.60E-086
		NC_011353	5315	5	YP_002268947.1	putative outer membrane autotransporter	1.80E-071	5.10E-082
					YP_002272121.1	hypothetical protein ECH74115_3895	4.20E-165	2.40E-079
					YP_002271604.1	putative autotransporter, IS5K-containing	1.70E-009	1.10E-061
					YP_002269864.1	pertactin family protein	2.30E-041	3.10E-059
					YP_002271639.1	putative outer membrane autotransporter adhesin	6.80E-005	3.20E-040
Escherichia coli O157:H7 str. EDL933	10	NC_002655	5298	9	NP_286049.1	putative beta-barrel outer membrane protein	1.80E-071	5.10E-082
					NP_289202.1	putative ATP-binding component of a transport system	1.20E-164	2.40E-079
					NP_287645.1	putative ATP-binding component of a transport system and adhesin protein	6.00E-003	9.50E-075
					NP_288772.1	putative ATP-binding component of a transport system	8.40E-005	1.10E-061
					NP_287154.1	putative adhesin	1.80E-053	3.10E-059
					NP_286746.1	putative adhesin	1.80E-053	3.10E-059
					NP_286112.1	putative structural protein (partial)	1.30E-004	1.70E-044
					NP_288807.1	adhesin	6.60E-005	3.20E-040
					NP_287760.1	putative outer membrane protein	8.30E-004	2.10E-027
		NC_007414	99	1	YP_325580.1	putative exoprotein-precursor	0.00E+000	6.60E-086
Escherichia coli O157:H7 str. Sakai	9	NC_002128	85	1	NP_052685.1	EspP	0.00E+000	6.60E-086
		NC_002695	5229	8	NP_308389.1	AidA-I adhesin-like protein	1.80E-071	5.10E-082
					NP_311542.1	ATP-binding component of a transport system	4.20E-165	2.40E-079
					NP_310143.1	ATP-binding component of a transport system and adhesin protein	6.00E-003	9.50E-075
					NP_311108.1	ATP-binding component of a transport system	8.40E-005	1.10E-061
					NP_309423.1	AidA-I	2.30E-041	3.10E-059
					NP_308451.1	flagellin structural protein	1.20E-004	6.00E-045
					NP_311143.1	adhesin	6.60E-005	3.20E-040
					NP_310034.1	BigB-like protein	9.40E-004	2.10E-027
Escherichia coli O157:H7 str. TW14359	7	NC_013008	5255	6	YP_003076326.1	putative beta-barrel outer membrane protein	1.80E-071	5.10E-082
					YP_003079435.1	adhesin-like autotransporter	4.20E-165	2.40E-079
					YP_003078928.1	putative autotransporter outer membrane protein	1.70E-009	1.10E-061
					YP_003077239.1	calcium-binding autotransporter	2.30E-041	3.10E-059
					YP_003078961.1	adhesin	6.80E-005	3.20E-040
					YP_003077786.1	autotransporter-1 (AT-1) family protein	8.10E-004	2.10E-027
		NC_013010	110	1	YP_003082230.1	putative exoprotein-precursor	0.00E+000	6.60E-086

Escherichia coli O26:H11 str. 11368	9	NC_013361	5364	9	YP_003227435.1	AidA-I adhesin-like protein	2.60E-070	6.60E-082
					YP_003228721.1	AidA-I adhesin-like protein	1.60E-004	8.10E-064
					YP_003228395.1	putative AidA-I adhesin-like protein	1.80E-053	3.10E-059
					YP_003232352.1	adhesin AIDA-I precursor	3.90E-112	1.20E-058
					YP_003229866.1	AidA-I adhesin-like protein	1.60E-112	1.20E-058
					YP_003230356.1	putative AidA-I adhesin-like protein	2.00E-054	9.20E-058
					YP_003230174.1	adhesin YfaL	5.50E-005	1.60E-043
					YP_003228754.1	putative adhesin	8.60E-005	1.70E-029
					YP_003230070.1	putative autotransporter outer membrane protein	7.10E-004	1.00E-008
Escherichia coli O55:H7 str. CB9615	10	NC_013941	5014	10	YP_003498036.1	AidA-I adhesin-like protein	1.80E-071	5.10E-082
					YP_003499005.1	putative ATP-binding component of a transport system	6.10E-127	1.10E-080
					YP_003499431.1	outer membrane autotransporter barrel domain protein precursor	6.60E-003	1.50E-072
					YP_003500261.1	putative autotransporter IS5K-containing	1.60E-009	1.10E-061
					YP_003498028.1	putative autotransporter	3.00E-004	1.40E-047
					YP_003501136.1	Type V secretory pathway, adhesin AidA	2.60E-046	1.00E-046
					YP_003498095.1	putative flagellin structural protein	1.30E-004	6.00E-045
					YP_003500295.1	putative ATP-binding component of a transport system	6.90E-005	3.20E-040
					YP_003499044.1	putative outer membrane autotransporter	8.90E-005	2.40E-028
					YP_003499324.1	hypothetical protein G2583_1763	8.20E-004	2.10E-027
Escherichia coli S88	6	NC_011742	4692	6	YP_002390114.1	Vacuolating autotransporter toxin	0.00E+000	2.90E-078
					YP_002394365.1	antigen 43 precursor (AG43) (Fluffing protein)	1.10E-052	7.00E-058
					YP_002390144.1	adhesin; outer membrane autotransporter barrel	2.40E-004	1.30E-047
					YP_002390196.1	flagellin-like structural protein	1.10E-004	4.80E-045
					YP_002392063.1	adhesin	5.60E-010	2.60E-041
					YP_002391254.1	autotransported outer membrane protein involved in cell adhesion	2.00E-004	9.10E-023
Escherichia coli SE11	7	NC_011415	4679	7	YP_002292875.1	putative lipoprotein/autotransporter domain-containing protein	6.50E-052	2.80E-074
					YP_002292490.1	putative autotransporter	1.40E-004	1.60E-064
					YP_002291602.1	putative autotransporter	2.70E-004	8.50E-048
					YP_002291668.1	putative autotransporter	1.60E-004	4.80E-045
					YP_002293769.1	adhesin	6.20E-005	2.70E-041
					YP_002292526.1	putative autotransporter	8.40E-005	1.30E-029
					YP_002293734.1	putative autotransporter	6.60E-004	1.30E-008
Escherichia coli SMS-3-5	8	NC_010498	4743	8	YP_001744804.1	hypothetical protein EcSMS35_2773	1.80E-153	1.90E-079
					YP_001744032.1	autotransporter (AT) family porin	1.30E-004	2.00E-066
					YP_001744553.1	outer membrane autotransporter	1.70E-033	4.60E-065
					YP_001745886.1	outer membrane autotransporter	4.10E-222	8.00E-060
					YP_001744298.1	antigen 43	2.80E-054	6.80E-053
					YP_001742505.1	outer membrane autotransporter	7.90E-004	5.20E-045
					YP_001744429.1	outer membrane autotransporter	2.40E-006	5.10E-042
					YP_001743821.1	putative autotransporter	1.70E-004	1.20E-027
Escherichia coli str. K-12 substr. DH10B	5	NC_010473	4126	5	YP_001730497.1	hypothetical protein ECDH10B_1640	6.30E-003	7.60E-074
					YP_001730949.1	CP4-44 prophage; antigen 43 (Ag43) phase-variable biofilm formation autotransporter	4.70E-115	5.20E-061
					YP_001731131.1	autotransporter outer membrane protein	1.00E-004	2.50E-059
					YP_001731171.1	adhesin	6.10E-005	5.90E-041
					YP_001730150.1	adhesin	9.20E-005	5.40E-031
Escherichia coli str. K-12 substr. MG1655	4	NC_000913	4145	4	NP_417134.2	adhesin-like autotransporter	1.40E-167	8.20E-082
					YP_026164.1	CP4-44 prophage; antigen 43 (Ag43) phase-variable biofilm formation autotransporter	4.70E-115	5.20E-061
					NP_416736.1	adhesin	6.10E-005	5.90E-041

					NP_415720.1	predicted adhesin	9.20E-005	5.40E-031
Escherichia coli str. K-12 substr. W3110	5	AC_000091	4226	5	AP_003226.1	adhesin-like autotransporter	1.40E-167	8.20E-082
					AP_002599.1	antigen 43 (Ag43) phase-variable biofilm formation autotransporter	4.70E-115	5.20E-061
					AP_002787.1	predicted autotransporter outer membrane protein	1.00E-004	2.50E-059
					AP_002830.1	adhesin	6.10E-005	5.90E-041
					AP_001827.1	predicted adhesin	9.20E-005	5.40E-031
Escherichia coli UMN026	11	NC_011751	4825	11	YP_002414040.1	Serine protease precursor (Secreted autotransporter toxin)	0.00E+000	1.20E-085
					YP_002413670.1	adhesin-like autotransporter	1.30E-156	1.30E-079
					YP_002412513.1	putative lipoprotein	2.20E-050	1.40E-071
					YP_002412204.1	hypothetical protein ECUMN_1459	2.00E-003	4.40E-067
					YP_002413240.1	putative autotransporter outer membrane protein; type V secretion	8.30E-005	2.40E-061
					YP_002415461.1	antigen 43 (Ag43) phase-variable biofilm formation autotransporter; CP4-44 prophage	2.30E-107	1.30E-056
					YP_002414067.1	antigen 43 (Ag43) phase-variable biofilm formation autotransporter; CP4-44 prophage	2.40E-085	8.70E-054
					YP_002411173.1	Putative flagellin-like structural protein similar to yaiT; putative exported protein	1.30E-004	4.80E-045
					YP_002413282.1	adhesin	5.70E-005	2.90E-042
					YP_002412243.1	putative adhesin; putative autotransporter	8.60E-005	7.20E-031
					YP_002412393.1	putative autotransported outer membrane protein involved in cell adhesion	9.50E-005	7.50E-028
Escherichia coli UTI89	5	NC_007946	5021	5	YP_540153.1	putative autotransporter	2.30E-055	1.70E-057
					YP_539366.1	hypothetical protein UTI89_C0334	2.40E-004	1.30E-047
					YP_539421.1	putative structural protein	1.10E-004	4.80E-045
					YP_541512.1	adhesin	5.60E-010	2.60E-041
					YP_540636.1	EntS/YbdA MFS transporter	1.90E-004	5.30E-023
